# Integrated AHP-TOPSIS under a Fuzzy Environment for the Selection of Waste-To-Energy Technologies in Ghana: A Performance Analysis and Socio-Enviro-Economic Feasibility Study

**DOI:** 10.3390/ijerph19148428

**Published:** 2022-07-10

**Authors:** Sandylove Afrane, Jeffrey Dankwa Ampah, Ephraim Bonah Agyekum, Prince Oppong Amoh, Abdulfatah Abdu Yusuf, Islam Md Rizwanul Fattah, Ebenezer Agbozo, Elmazeg Elgamli, Mokhtar Shouran, Guozhu Mao, Salah Kamel

**Affiliations:** 1 School of Environmental Science and Engineering, Tianjin University, Tianjin 300072, China; sandfran20@gmail.com (S.A.); jeffampah@live.com (J.D.A.); 2 Department of Nuclear and Renewable Energy, Ural Federal University Named after the First President of Russia Boris Yeltsin, 19 Mira Street, 620002 Ekaterinburg, Russia; agyekumephraim@yahoo.com or; 3 Environmental Engineering Department, Egypt-Japan University of Science and Technology, New Borg El Arab 5221241, Egypt; amohprinceoppong@gmail.com; 4 Department of Mechanical and Automobile Engineering, Sharda University, Knowledge Park III, Greater Noida 201310, UP, India; abdulfatahabduyusuf@gmail.com; 5 Centre for Green Technology, Faculty of Engineering and IT, University of Technology Sydney, Ultimo, NSW 2007, Australia; IslamMdRizwanul.Fattah@uts.edu.au; 6 Department of Big Data Analytics and Methods of Video Analysis, Ural Federal University Named after the First President of 10 Russia Boris Yeltsin, 19 Mira Street, 620002 Ekaterinburg, Russia; eagbozo@urfu.ru; 7 Wolfson Centre for Magnetics, Cardiff University, Cardiff CF24 3AA, UK; shouranma@cardiff.ac.uk; 8 Electrical Engineering Department, Faculty of Engineering, Aswan University, Aswan 81542, Egypt; skamel@aswu.edu.eg

**Keywords:** AHP, circular economy, fuzzy TOPSIS, multi-criteria decision-making (MCDM), municipal solid waste (MSW), waste-to-energy (WtE), zero waste

## Abstract

Energy recovery from waste presents a promising alternative for several countries, including Ghana, which has struggled with unsustainable waste treatment methods and an inadequate power supply for several decades. The current study adopts a comprehensive multi-criteria decision-making approach for the selection of an optimal waste-to-energy (WtE) technology for implementation in Ghana. Four WtE technologies are evaluated against twelve selection criteria. An integrated AHP-fuzzy TOPSIS method is applied to estimate the criteria’s weights and rank the WtE alternatives. From the AHP results, technical criteria obtained the highest priority weight, while social criteria emerged as the least important in the selection process. The overall ranking order of WtE technologies obtained by fuzzy TOPSIS is as follows: anaerobic digestion *>* gasification *>* pyrolysis *>* plasma gasification. The sensitivity analysis indicates highly consistent and sturdy results regarding the optimal selection. This study recommends adopting a hybrid system of anaerobic digestion and gasification technologies, as this offers a well-balanced system under all of the evaluation criteria compared to the standalone systems. The results of the current study may help the government of Ghana and other prospective investors select a suitable WtE technology, and could serve as an index system for future WtE research in Ghana.

## 1. Introduction

Energy is a critical element in human existence, and is essential to a society’s sustainable economic growth. Globally, fossil fuels have been the primary source of electrical generation. Based on the most recent Statistical Review of World Energy, fossil fuels account for around 84% of global electricity generation [1]. However, the over-reliance on conventional fossil fuels presents a substantial challenge owing to dwindling reserves, which cause an escalation of fuel prices as well as emissions of greenhouse gases and other pollutants, all of which contribute significantly to global warming [2]. Renewable energy development will play a key role in attaining the United Nations 2030 Agenda for Sustainable Development and the Paris Agreement on Climate Change. For this reason, most developed countries have made huge investments in conversion technologies for renewable energy resources (solar, wind, hydro, biomass, geothermal, nuclear, ocean, wave, and tidal) to alleviate potential energy crises and protect the environment [3]. One disadvantage of using alternative energy sources such as solar, wind, and hydro is their inconsistency in supply, as they are mainly weather-dependent. When utilized as the main feedstock for power production, municipal solid waste (MSW) has proven to be more reliable in the generation of constant and large amounts of energy while mitigating environmental problems [4].

MSW management has become a topic of interest in most studies due to its numerous socioeconomic and environmental benefits. The generation and management of MSW has evolved dramatically over the last few decades. Globally, the trend of solid-waste management has begun to shift from uncontrolled landfilling to more sustainable options like recycling, composting, and energy recovery. The rates of recycling and energy recovery have increased from 6% and 0% of MSW generated in 1960 to around 32% and 12% in 2018, respectively. On the other hand, landfilling decreased significantly, from 94% of the amount generated in 1960 to 50% of the amount generated in 2018 [5]. It has been realized in recent years that instead of being a ‘burden’, MSW could be one of the world’s potential resources that have not yet been fully exploited. The stored energy in MSW may be recovered to generate heat and/or power via various biochemical or thermochemical processes based on the variable composition of the waste and its moisture level, which are dependent on local demographics and culture [6]. The waste-to-energy (WtE) technology supply chain offers a way to concurrently solve problems such as energy consumption, waste management, and greenhouse gas emissions, resulting in a circular economy system [7]. Therefore, regardless of the recent economic downturn, the worldwide market for WtE technologies has expanded significantly, and has the potential to generate approximately 13 GW of energy according to an estimate by the International Renewable Energy Agency [8]. The feasibility of different WtE technologies is clear in developed countries, owing to technology development, sufficient technical and analytical data, and government backing [9].

Conversely, in most developing countries, including Ghana, waste management is presently dependent on a linear economy, where materials are manufactured, utilized, and dumped into landfill [10]. The dramatic upsurge in the quantity of MSW at nonengineered landfills and open dumps has led to various environmental, socioeconomic and health problems. As a result, the government of Ghana is planning to adopt sustainable waste management solutions, which includes available WtE technologies to redirect waste from landfills and reduce environmental pollution, whiles simultaneously providing an additional source of renewable energy to tackle the country’s energy issues. Considering the increasing number of new technological options, selecting the optimal WtE alternative for the country is complicated due to the required comprehensive decision-making process [11]. As such, selecting the most feasible alternative against all evaluation indicators necessitates the involvement of several experts and stakeholders, in order to reach a trade-off resolution. This current work, therefore, adds to the existing literature by presenting a multicriteria decision framework for the evaluation of many waste-to-energy alternatives simultaneously based on a variety of technical, economic, environmental, and social indicators.

### 1.1. Background: Ghana’s Energy Profile

As one of the fastest developing economies in Africa, Ghana’s electricity demand has increased steadily over the years due to economic growth, urbanization, industrialization, and rural electrification [12]. Ghana’s total primary energy supply in 2019 was 11,149 Ktoe, rising by 2.7% from 10,852 Ktoe in 2018 [13]. As of 2019, electricity accounted for 15% of total primary energy consumption [13]. From 2005 to 2015, economic growth—as defined by the gross domestic product (GDP)—averaged 4.5% per year, while electricity consumption averaged 9.5% per year [12,14]. The peak demand for the system in 2020 was 3090 MW, up to 10.2% above the highest demand in 2019 [14]. However, the growth in this energy demand is not parallel to the country’s installed capacity. The total installed electricity generation capacity increased from 5172 MW in 2019 to 5288 MW in 2020 [14]. Put into perspective, it is estimated that the 10.2% increase in Ghana’s energy demand growth annually only correlates to a 2.2% increase in installed capacity. The imbalance between energy demand and supply has caused severe challenges, including frequent power outages and, in extreme circumstances, total blackouts [15]. This power crisis has badly affected the country’s GDP. Furthermore, Ghana’s objective of universal access to power by 2020 was not met, as the current electricity access rate across the country is at 83.5% [16]. These electricity-related problems have put enormous pressure on the government and other stakeholders to remedy the situation. 

**Figure 1:**
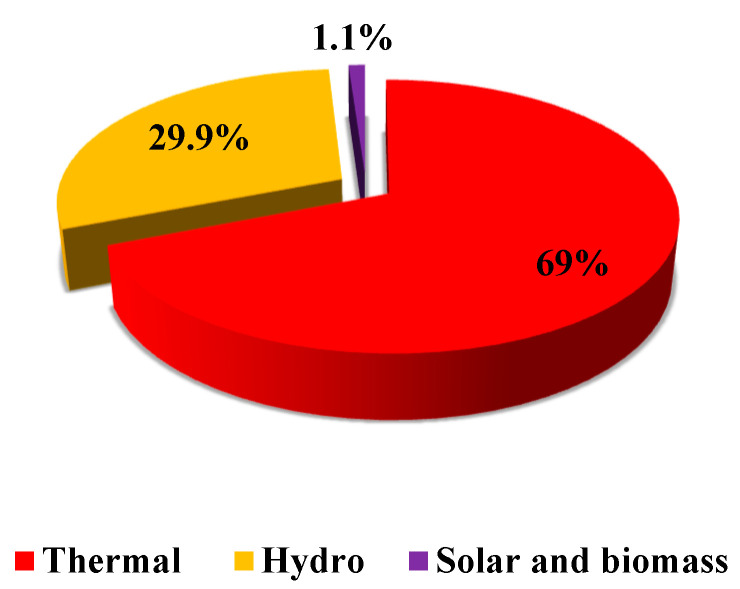
Share of Ghana’s installed power capacity by source in 2020 [14].

The government of Ghana has identified renewable energy as one of the promising alternatives to contribute to the energy supply mix while reducing the environmental menaces of energy production [17]. A target was set in 2016, according to the renewable energy act, to increase renewable electricity production by 10% by the end of 2020 [18]. Despite various interventions and goals, the country could only meet 0.8% of its 10% renewable energy targets as of December 2019. Ghana’s electricity mix has mainly remained constant, with conventional thermal plants contributing 69% of the total installed capacity, and with hydro accounting for 29.9%, while other renewable energy sources (biomass and solar) accounted for only 1.1%, as shown in Figure 1 [14].

This necessitated the creation of the Renewable Energy Master Plan (REMP) in 2019 to develop a roadmap focused on socioeconomic progress and reducing the negative effects of climate change by 2030 [19]. Electricity generation from MSW is expected to reach 50.1 MW of utility-scale power in 2030 (Table 1). According to the masterplan, some merits for the integration of WtE in Ghana’s energy mix include: (1) boosting the power generation capacity via the use of less-fluctuating conversion technologies, (2) fixed electricity for industrial applications, and (3) providing employment opportunities [19].

### 1.2. Background: The MSW Situation in Ghana

**Figure 2:**
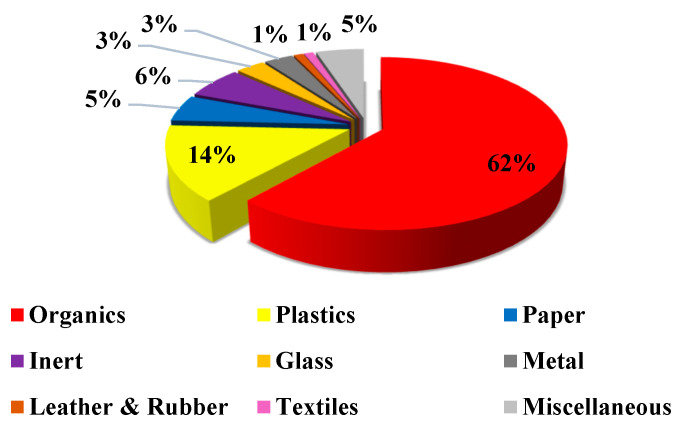
Estimated composition of MSW in Ghana [20].

Like most developing economies, waste generation in Ghana is increasing rapidly, especially in urban areas, due to rapidly growing populations, urbanization, and industrialization. An estimated 12,710 tons of waste is generated daily, which translates into 0.47 kg/person/day [20]. MSW constitutes the largest portion of the total waste generated in Ghana. As represented in Figure 2, organic materials constitute about 62% of household MSW in Ghana [20]. Factors such as the waste type and volume are significant in the adoption of an optimal energy recovery method and for a cost–benefit analysis of the project.

Effective waste collection in urban areas accounts for only 52% of the total waste generated, with the remaining either being burned by households, dumped indiscriminately in the open, and/or disposed of in water bodies [21]. Despite the availability of recyclables, which constitute about 22% of the waste stream, waste management options such as recycling and composting are faced with extra operational costs, as the source sorting of MSW is not widely practiced in Ghana [21]. It is incumbent on appropriate governmental agencies to enforce and implement sanitation laws to help curb waste management issues. MSW as a resource for energy production through advanced treatment technologies has received much attention in various parts of the world. However, the situation is different in Ghana and other African countries due to technical, environmental and socioeconomic management system challenges. Several feasibility studies have indicated that given the enormous quantity and composition of waste produced, electricity production from MSW is very viable in Ghana [21,22,23].

## 2. Literature Review

In an attempt to lessen the problem-solving time and complexities and boost productivity, several approaches have received particular attention in the last few decades, including machine learning [24,25] and multicriteria decision methods (MCDM) [26,27]. The selection of a feasible WtE technology amongst various alternatives is a multi-criteria problem, and has over the years been tackled with various MCDM techniques in the existing literature. MCDM techniques have been used to solve a variety of energy-related issues, such as energy planning and management, the distribution of energy resources, energy policy, and structural energy management [28]. A bibliometric review of MCDM in sustainable energy planning revealed about 142 papers discussed and analysed in terms of applicability and methodologies used from 1998 to 2019 [29]. A large number of these papers have focused on the planning and selection of WtE alternatives. Table 2 presents previous studies that have applied multicriteria decision-making models to select optimal WtE alternatives in various countries/regions. The selection of a specific technology for energy recovery from MSW is based on critical evaluation criteria such as environmental friendliness, techno-economic feasibility, geographical conditions, sustainability, and socio-political benefits. Based on the specific objectives of the research, various studies have considered different sustainability indicators for the evaluation of the feasibility of a WtE technology. While most of these studies have considered the four common criteria (technical, economic, environmental, and social criteria) in the WtE technology selection process, others have focused on only one or two criteria. For instance, Milutinović et al. [30] only considered environmental indicators, Fernández-González et al. [31] focused on economic and environmental criteria, and Afrane et al. [32] evaluated the techno-economic feasibility of WtE alternatives.

In model application, AHP has been frequently utilized to evaluate WtE technologies and determine the development and investment priority. Yap and Nixon [11] applied AHP to assess the trade-offs between the benefits, opportunities, costs, and risks of various WtE technologies in India and the UK. Their results showed that gasification is the most suitable WtE alternative for implementation in the UK, whereas anaerobic digestion is the optimal alternative for India. Using the AHP method, Qazi et al. [33] also selected the best WtE alternative to enhance Oman’s waste management system. Based on the criteria selected for evaluation, this study suggested that anaerobic digestion is the best alternative for Oman. Abdallah et al. [34] implemented the AHP to rank the importance of the selected criteria in the decision-making process of optimal WtE selection for the Kafr El-Sheikh Governorate in Egypt. Energy production obtained the highest weight in the multicriteria assessment, and anaerobic digestion with recycling was ranked as the most suitable WtE strategy in this study.

Other methods like TOPSIS, VIKOR, and DEMATEL have also been applied to solve multi-objective problems related to WtE selection. Alao et al. [35] employed the TOPSIS model to identify anaerobic digestion as the best technique for electricity generation from waste, ahead of pyrolysis, incineration, and landfill gas recovery in Lagos, Nigeria. Interval-valued fuzzy DEMATEL was developed and used by Wang et al. [36] to identify and prioritize MSW treatment technologies for Chongqing, China. In a decreasing order of suitability, the developed model arranged the technologies as follows: anaerobic digestion, gasification, incineration, and landfill gas recovery.

The use of integrated MCDM models in sustainable energy planning has also increased considerably in recent years. In WtE selection, Shah et al. [37] integrated ANP, VIKOR, and DEMATEL with fuzzy theory to evaluate WtE technologies under Pakistani conditions based on the three dimensions of the energy trilemma. Gasification emerged as the most favorable technology for investment, while torrefaction technology was considered the least favourable. Furthermore, in Iran, gasification, anaerobic digestion, pyrolysis, and incineration were ranked with ANP, DEMATEL, and SAW under fuzzy conditions by Fetanat et al. [38]. The results confirmed anaerobic digestion and incineration as the best and worst technologies for consideration in Iran, respectively. By using Fuzzy AHP for the criteria weights and TOPSIS for the ranking of alternatives, Islam et al. [39] evaluated potential WtE technologies in order of preference. Gasification and co-combustion were the two most promising technologies for the conversion of waste to energy in Dhaka, Bangladesh.

**Table 2:** MCDM models applied to WtE-related studies in various countries/regions.

Study Area	WtE Alternatives	MCDM Models	Evaluation Criteria	Ref.
India, UK	INC, GAS, AD, LFGTE	AHP	Technical, Economic, Environmental, Social	[11]
India	LFGTE, AD, INC, palletisation, GAS	AHP	Technical, Financial Environmental, Risk	[6]
Pakistan	INC, GAS, PYR, PT, AD Torrefaction, Fermentation,	Fuzzy ANP, Fuzzy VIKOR, DEMATEL	Energy security and equity, Environmental sustainability	[37]
Nigeria	INC, AD, LFGTE, PYR	TOPSIS	Technical, Economic, Environmental	[35]
South Africa	INC, GAS, AD, PYR	Fuzzy-AHP, Fuzzy-Entropy, Fuzzy MULTIMOORA	Technical, Economic, Environmental, Social	[40]
Russia	LFGTE, INC, AD, RDF	AHP	Technical, Economic, Environmental, Social	[41]
Bangladesh	Co-combustion, INC, GAS, PYR	Fuzzy AHP, TOPSIS	Technical, Economic, Environmental, Social	[39]
Iran	INC, GAS, AD, PYR	Fuzzy DEMATEL, ANP, SAW	Energy, Rights, Social, Environmental	[38]
Oman	INC, AD, GAS, PYR, PAG, TDP, HTC, Fermentation	AHP	Technical, Economic, Environmental, Social	[33]
China	INC, GAS, AD, LFGTE	DEMATEL	Technical, Economic, Environmental, Social	[36]
Egypt	INC, AD, LFGTE	AHP	Energy, Economic, Environmental	[34]
Spain	AD, SRF, GAS, INC	AHP	Economic, Environmental	[31]
Serbia	INC, AD, LFGTE	AHP	Environmental	[30]

INC: Incineration; AD: Anaerobic digestion; GAS: Gasification; LFGTE: Landfill gas-to-energy; PYR: Pyrolysis; PT: Plasma treatment; RDF: Refused derived fuel; PAG: Plasma arc gasification; TDP: Thermal de-polymerization; HTC: Hydrothermal carbonization; SRF: Solid recovered fuels; AHP: Analytic hierarchy process; ANP: Analytic network process; VIKOR: Vlse Kriterijumska Optimizacija Kompromisno Resenje; DEMATEL: Decision-making trail and evaluation laboratory; TOPSIS: Technique for order preference by similarity to ideal solutions; MOORA: Multi-objective optimization by ration analysis; SAW: Simple additive weighting.

In the past, few studies have been conducted to thoroughly evaluate the potential and viability of WtE implementation in Ghana. A recent study investigating the energy recovery potential of MSW in one of Ghana’s major cities, Kumasi, found that 1 m^3^ of biogas produced from MSW in Kumasi may provide up to 36 MJ of energy, which is equivalent to 10 kW/h of electricity [23]. Another piece of research by Ofori-Boateng et al. [21] suggested that given the MSW characteristics in Ghana, about 4.5 million tons of waste may produce around 2 GWh of energy per year through regulated combustion, and 1.0–1.5 GWh of electricity per year through controlled landfilling. Findings from Kemausuor et al. [22] also revealed that Ghana’s power demand might be met to some degree by the utilization of agricultural leftovers, animal wastes, wood residues, and MSW to generate about 96 PJ in 2700 Mm^3^ of biogas. From a techno-economic standpoint, Osei-Appiah and Dioha [42] established that while biochemical technologies like anaerobic digestion have the advantage of cost-effectiveness, the thermochemical processes such as gasification and pyrolysis have a higher efficiency for energy conversion. Although these studies make important additions to the field, none of them addresses the issue of which WtE technology is optimal for implementation in Ghana based on a variety of evaluation indicators. According to the assessment of these works of literature, no WtE alternative has a full benefit over the other and operates distinctively in economic, environmental, technical, and social dimensions. This then makes it challenging for decision-makers and stakeholders to make a definitive choice regarding the optimal WtE alternative for implementation in Ghana. As a result, it is essential to contribute to the current body of literature a study that provides a framework for the ideal identification of the optimal WtE alternative for power generation in Ghana while simultaneously addressing all of the performance indicators.

Similar to the studies from different countries/regions, as reviewed above, the use of MCDM for WtE technology selection has been studied for Ghana. To the best of our knowledge, only two studies—i.e., [32,43]—have attempted similar objectives to that of the current work. Agbejule et al. [43] used the AHP method to prioritize four technologies (landfill biogas, incineration, anaerobic digestion, and aerobic composting) for investment in Ghana based on technical, environmental, economic, and social criteria.

Despite their study’s key contribution and significance, the sole use of the AHP method has often been criticized. The conventional AHP is a subjective method that depends on experts’ opinions. In addition, the random adjustment of the consistency ratio until the desired value is obtained is also subjective. Subjective decisions are prone to ambiguity, vagueness, and bias, which makes the reliability of the sole use of the AHP method questionable to some extent. The second study is by Afrane et al. [32], where the issue of expert subjectivity was addressed using a fuzzy approach by the TOPSIS method. However, their study only considered the techno-economic aspects of the WtE technologies. It is important to consider the technologies’ environmental and social feasibilities for sustainability purposes. Furthermore, both studies have only considered individual MCDM methods, but there is convincing evidence in the literature suggesting the superiority of hybrid MCDM methods [44]. For these reasons, the current study improves upon the existing studies on Ghana’s WtE technology selection by proposing a hybrid AHP-fuzzy TOPSIS method. As far as we are concerned, this will be the first work to use this hybrid MCDM method to select WtE technologies.

In summary, the primary aim of the current research is to assess the feasibility of WtE technologies for implementation in Ghana through a multi-criteria decision analysis of the chosen WtE alternatives against a set of defined criteria. This offers an initial evaluation of the significance of several variables affecting the selection of WtE technologies from the standpoint of experts and stakeholders in the waste management and energy recovery sector. The goal is to ascertain the most technically feasible, most cost-effective, least polluting, and most socially acceptable waste-to-energy conversion technology among other selected alternatives for the generation of electricity from MSW in Ghana. The particular questions to be answered are:

- What are the technical, economic, environmental, and social implications of the WtE technologies chosen?
- What are the relative weights of several indicators that influence the choice of WtE alternatives in the Ghanaian context?
- In light of the experts’ and stakeholders’ interests, which WtE technology is most suited for the conditions of Ghana?

This research is useful in providing information on feasible treatment technologies for the management of the country’s waste through energy recovery. The present study’s findings may help the government of Ghana and other prospective investors choose a suitable WtE technology that offers the greatest benefit under multiple criteria for minimizing Ghana’s electricity and waste management problem. Additionally, assessing the most important sustainability criteria could serve as an index system for future waste-to-energy research in Ghana.

## 3. Description of the Existing Waste-To-Energy Technologies

Technologies that can efficiently recover energy from waste with little to no environmental damage have been considered as alternatives for conventional fossil fuel generation [45]. The conversion pathways utilized in waste-to-energy technologies may be classified into two main categories: thermochemical and biochemical. The thermochemical pathway involves the conversion of MSW feedstock to energy (electricity, heat, and value-added products) under high temperatures. It is most commonly utilized for dry waste with a larger proportion of nonbiodegradable material (a low water content). Combustion (incineration), gasification, and pyrolysis are the most common technologies under thermochemical conversion of MSW. Biochemical processes are optimal for wastes with a high proportion of biodegradable organic materials and a high moisture/water content that promotes microbial activity. Anaerobic digestion (AD) and methane recovery from a controlled environment in landfills are the most common technologies under the biochemical pathway. The subsequent sub-sections describe the various technologies in terms of their efficiency and impact on global power systems.

The WtE technologies considered for this analysis include AD, conventional gasification, pyrolysis, and plasma gasification. The justification for choosing these technologies is based on two primary considerations: firstly, the technical maturity of the WtE technologies in Ghana; secondly, the availability and accessibility of data on these technologies to make an informed decision. However, for comparative purposes, incineration and landfill gas recovery technologies are reviewed alongside the selected WtE technologies in this section.

### 3.1. Combustion (Incineration)

The incineration of waste is the most common WtE technology integrated into waste management systems worldwide. It is the oxidation of combustible waste constituents, and is utilized for a diverse variety of MSW. The efficiency of the incineration process is estimated to be about 25–30%, and it is mainly influenced by the constituents of the waste stream, particularly for MSW, which may be extremely diverse [46,47]. In general, there are two types of incineration for energy recovery: mass-burn and refuse-derived fuels (RDF). In mass burn technology, MSW is burnt completely, without any pre-processing. In RDF plants, recycling and non-combustible elements are sorted, and the remaining waste is shredded, dried, and compressed to generate fuel with relatively homogenous characteristics. Pre-processing is performed in RDF to obtain a higher heating value. Even though RDF has proven to be a more efficient technology, mass-burn is a more common technology used worldwide [48]. MSW is combusted at high temperatures in a specially constructed chamber with a continuous air supply to guarantee turbulence and the full combustion of the elements to their stable and original molecular states. The end products of the combustion are mainly hot combusted gases like carbon dioxide, nitrogen, oxygen, flue gas, and non-combustible elements [49].

The incineration of MSW operating at an uncontrollably high temperature can produce a net energy of about 544 kWh/ton MSW, but this process is more environmentally damaging. Combusting MSW at uncontrolled temperatures produces chlorinated dibenzo-p-dioxins and corrosive gases that could destroy the steam pipes and cause health-related problems. Dioxins, particulate matter, sulfur dioxide, hydrochloric acid, and heavy metals are all possible contaminants in flue gases. In previous years, the environmental impact of dioxins was one of the most crucial challenges related to MSW incineration [50]. In recent years, however, most modern incinerators have used a sophisticated pollutant/emissions control system designed according to developed countries’ stringent regulations to reduce air pollutants and emit virtually no dioxins [51].

### 3.2. Gasification

Gasification is a process in which organic materials are partly oxidized at high temperatures (usually 500–1800 °C) with reduced oxygen [52]. The gasification process is influenced by the temperature, pressure, and oxygen concentrations. The gaseous product obtained from this process is known as synthetic gas (syngas), and it mainly consists of hydrogen, carbon monoxide, carbon dioxide, and methane, together with heat (used to generate power and process heat) [47]. The gasification agent (air, oxygen, or steam), operating temperature and pressure of the gasifier, and feed properties all impact the end products’ chemical composition and energy content. The syngas obtained from the gasification process is very high in calorific value, and may be transformed into other valuable products such as alternative transport fuels, natural gas replacements, and fertilizers, as opposed to just heat and electricity from the incineration process in a WtE plant [53,54].

Biological waste, sewage sludge, industrial waste, and wood waste have previously been treated using gasification methods. However, in recent years, the gasification of MSW is increasingly gaining popularity. Several gasification technologies for the cogeneration of heat and electricity from syngas have been developed during the previous two decades and are now commercially accessible [55]. Gasification facilities share environmental problems similar to those associated with mass-burn incinerators, including water pollution, air pollution, ash disposal, and other byproducts. During gasification, tars, alkaline compounds, halogens, and heavy metals are released and can cause environmental and operational problems.

### 3.3. Plasma Gasification

Plasma gasification uses an electrically driven plasma torch to volatize waste and organic materials with a regulated infusion of oxygen [56]. The organic component of the waste stream is processed into synthetic gas (syngas), while the inorganic portion is processed into an inert vitrified glass that may be utilized for a variety of construction products [57]. Plasma is the fourth state of matter, consisting of heated ionized gases generated by an electrical discharge at very high temperatures (2000–5000 °C) [58]. The energy contained in a plasma enables the utilization of low-energy fuels, such as domestic and industrial waste, which are often incapable of supporting their own gasification without extra fuel. The synthesis gas may be utilized to generate efficient power and/or heat, and second-generation liquid biofuels [55]. Typically, no ash remains after the process to be dumped in a landfill. The characteristics of the waste stream, however, may have an impact on the effectiveness of the gasification process. Waste with a high proportion of inorganic materials, such as metals and construction waste, may provide less syngas and more slag.

### 3.4. Pyrolysis

Pyrolysis is the thermal breakdown of a substance in the absence of or with a restricted supply of an oxidizing agent (i.e., partial gasification) to produce the thermal energy needed for the process. Compared to gasification, relatively modest temperatures (400–900 °C, although typically less than 700 °C) are used [59]. Syngas is produced during pyrolysis, which is the thermal breakdown of carbon-based compounds using heat, rather than direct combustion. Particle matter, mercury, sulfur, and other pollutants are removed from the syngas. Pyrolysis gas, pyrolysis liquid, and solid coke are the three products produced, the proportions of which are highly dependent on the pyrolysis technique and reactor process parameters [60].

Pyrolysis processes are classified under three main categories; slow pyrolysis, rapid (or flash) pyrolysis at high temperatures, and flash pyrolysis at low temperatures [61]. The most commonly used pyrolysis process among them in present times is flash pyrolysis. This is because the slow pyrolysis process requires a very long period of time (many hours) and yields bio-char as the primary product, while flash pyrolysis produces about 60% bio-oil in only a few seconds [61,62]. Most pyrolysis technologies are environmentally friendly ways of converting feedstock; however, if the right measures are not taken, they can cause adverse environmental and health issues. For instance, technologies that do not recycle their syngas, including small, homemade technologies or traditional systems, allow these gases to escape, which can double the carbon produced rather than being carbon neutral, partially from the use of fossil fuel and partially from avoiding the use of the produced fuel. Additionally, oil and tar contain heavy organic chemicals that are harmful to the environment.

### 3.5. Anaerobic Digestion (AD)

Anaerobic digestion is the breakdown of biodegradable material by microorganisms in the absence of oxygen. For the digesting process, special reactors are utilized, and within the reactors, certain variables—such as pH, moisture content, and temperature, among others—are regulated [63]. These parameters aim to create a suitable habitat for microorganisms, allowing them to multiply and accelerate the methane breakdown process. The bioconversion process is complicated and divided into four phases. The first step is hydrolysis, in which extracellular enzymes break down complex insoluble organic molecules like proteins, lipids, and carbohydrates into soluble organic materials such as sugars, amino acids, and fatty acids. Following this is the acidogenesis process, which involves the breakdown of hydrolysis products into acetate, hydrogen, and higher-molecular-weight volatile fatty acids. Acetogenesis is the third step, in which acidogenesis products are further processed into acetic acid, CO_2_, and hydrogen by acetate-forming bacteria. The last step of methanogenesis, in which methanogens may develop at low redox potentials using substrate-level or electron transport phosphorylation, generates biogas, a combination of gaseous molecules—mostly methane and carbon dioxide—through volatile fatty acid breakdown [64,65].

Biogas may be used to generate electricity, fuel (biomethane) for combustion engines, and space heating, water heating, and process heating. Over 90% of the energy available in the wastes is kept in the biogas as methane during the anaerobic conversion or fermentation of MSW, with the remainder being sludge [21]. Anaerobic digestion adds value to MSW while avoiding several negative effects connected with the natural breakdown process that happens in landfills, and allows the replacement of alternative fossil source materials. Most AD facilities around the globe are used for sewage sludge and animal manure, with MSW treatment being more complex and still under development.

### 3.6. Landfill Gas Recovery

Landfilling has been the only disposal technique capable of dealing with all of the materials in the solid waste stream, as well as the easiest and, in many cases, cheapest disposal option. The generation of landfill gas from a sanitary landfill plant is similar to anaerobic digestion, with the main difference being the operational control of the sanitary landfill [40]. Because of the rapid reaction pace combined with the temperature stability, biochemical breakdown in biogas reactors is better regulated. When organic waste is disposed of in a landfill, it emits landfill gas (LFG) as it degrades under anaerobic conditions. Instead of letting these gases enter the environment and contribute to global warming, landfill gas plants may collect them, isolate the methane, and burn them to produce electricity, heat, or both. Landfill gas typically comprises 50% methane and 50% carbon dioxide, with an energy level of 18–19 MJ/m^3^ [66]. In most cases, land scarcity and other threats to the environment brought on by air, water, and land pollution from effluent make landfilling, despite its low cost, unsustainable and unsuitable for treating MSW

## 4. Selection of Criteria and Sub-Criteria

The four WtE alternatives were chosen with the goal of integrating both the universal solution and certain new technologies. However, each of these alternatives has its benefits and disadvantages. As a result, choosing the optimal WtE technology among the four alternatives is challenging for decision-makers based on only one criterion or the other. There is, therefore, a need to integrate all of the criteria that have a relevant impact on the optimal selection. A thorough literature study on energy recovery from waste in general and the MSW and energy sectors in Ghana was performed to choose the criteria that would be utilized in assessing the technologies. Based on the literature review, the assessment criteria for the selection of WtE technologies may be classified in four major categories: technical, economic, environmental, and social aspects. The technical aspect concerns factors that affect energy production, such as conversion efficiency, power generation capacity, and technology maturity. The economic component is concerned with cost-related factors such as capital cost, O&M cost, and the cost of energy. The environmental component is concerned with the detrimental effect on the environment, such as carbon dioxide emissions and the production of hazardous residue. The social component defines the maximum of people’s social well-being, such as social acceptance and job creation. The various sub-criteria adopted for assessing the optimal WtE alternative based on the four main sustainability criteria are summarized and reviewed in Table 3.

## 5. Method

**Figure 3:**
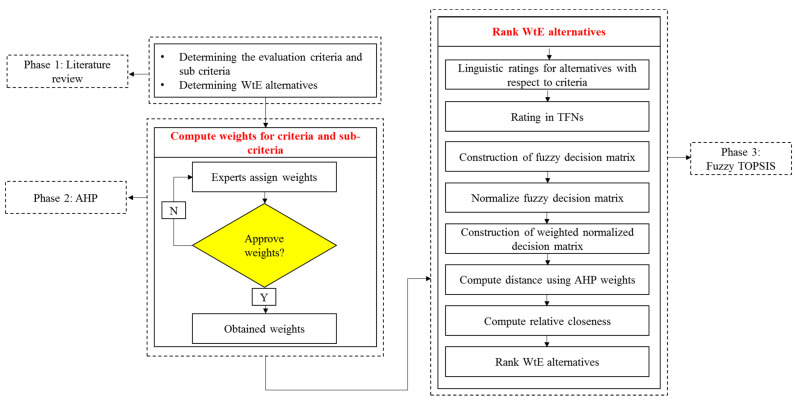
The process of selecting the optimal WtE alternative depicted in a methodological framework.

This research aims to evaluate the feasibility of various WtE technologies for implementation in Ghana, and to make an optimal selection against different indicators. To begin with, a thorough literature review was conducted on the various WtE technologies, the factors that affect their feasibility, and different MCDM techniques and their applications in sustainable energy planning and waste management strategies. A study of the different WtE alternatives led to the establishment of appropriate selection criteria for the decision-making process. This study’s criteria include a variety of technological, economic, environmental, and social aspects. The weights for the criteria might be qualitative, quantitative, or a combination of both. Quantitative or objective weights represent the characteristics of the possibilities available numerically, and they are best suited for studies for which adequate and easily accessible research data are plentiful. In contrast, qualitative or subjective weights are dependent on decision-makers’ views and judgments regarding the characteristics of the options, and thus are best suited for study areas with relatively scarce research data, such as Ghana. In order to achieve the intended results, the present research uses a qualitative decision-making method. A questionnaire is created and sent to ten experts with extensive expertise and background information on WtEs for developing nations with MSW characteristics and economies similar to Ghana’s. An integrated MCDM technique that combines AHP and fuzzy TOPSIS is used for the analysis. The AHP methodology is used to calculate the weights of the selection criteria and sub-criteria. The obtained weights are then used to rank the optimal WtE technology for investment in Ghana using the fuzzy TOPSIS method. Because expert judgments may be confusing, unclear, and imprecise, the subjective characteristics are represented numerically using both Saaty’s basic scale of absolute numbers and the fuzzy conversion scale. Finally, a sensitivity analysis is conducted to see how changing the experts’ original weights would affect the overall priority ranking of the WtE technologies. The decision framework used to identify the optimal WtE selection is shown in Figure 3.

### 5.1. Analytical Hierarchy Process

Saaty developed the AHP technique in the 1970s [67]. AHP enables researchers to break down their decision problems into a hierarchy of subproblems, each of which may be evaluated separately. It gives a decision-maker the freedom to quantify criteria and alternatives concerning the overall objective using numerical priority calculations. The AHP method relies on pair-wise comparisons of the specified criteria used to determine the weight for the evaluation of alternative performance ratings. In order to make pairwise comparisons easier, a decision hierarchy tree is built to provide the overall framework that determines the number of comparisons to perform. The criteria weights are assigned by questioning how significant criterion A is in relation to criterion B in terms of the main goal. Sub-criteria weights are also assigned by questioning how significant sub-criterion A1 is compared to sub-criterion A2 with regard to criteria A. Saaty [68] developed a relative measurement scale for pairwise comparison to determine what score or weight to provide, as shown in Table 4. It shows the intensities used to weigh a criterion and how they should be understood. Assigning a score of 1 can be interpreted as the two criteria being of equal importance; an intensity of 3 is assigned to mean that one criterion is slightly more important than the other, while assigning an intensity of 9 gives a strong indication that one criterion has the utmost importance over the other. Intensities 2, 4, 6, and 8 may be seen as intermediate levels utilized when the relative assessment is less apparent.

A pairwise comparison matrix is created for each comparison. Following the construction of the pairwise comparison matrices, the rankings are determined by computing a priority vector for each comparison matrix. There is a stringent consistency requirement to maintain the validity of preference patterns between the compared components. A consistency check is performed to ensure that the pairwise comparisons are properly assessed. In this study, the consistency check was performed using Saaty’s consistency ratio, as Saaty’s relative measurement scale is used for scoring and the consistency ratio is simpler to understand and use than the other techniques.

The main steps involved in the AHP technique are outlined simply below [69]:

Step 1: Identify the decision problem and determine its goal.

**Figure 4:**
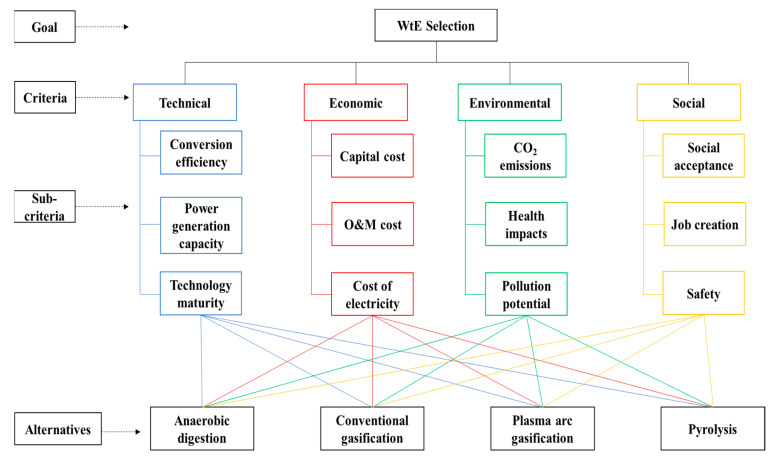
Hierarchical decision tree.

Step 2: Create a four-tiered hierarchical structure, as shown in Figure 4. The framework begins at the top (goal), then moves down to the intermediate orders (the criteria and sub-criteria), and finally to the bottom level (alternatives).

Step 3: Create a pairwise comparison matrix (n×n) using Saaty’s 1–9 basic scale of absolute numbers. The pairwise comparison matrices are defined by which item outperforms the other.

Step 4: Determine the significance of the pairwise comparison by creating a matrix of relative ranks for each hierarchy stage.

Step 5: Calculate the consistency index (*CI*) and consistency ratio (*CR*).

(1) $$CI=\frac{\lambda_{max}-n}{n-1},$$

where $\lambda_{max}$ is the eigenvalue and *n* is the number of criteria;

(2) $$CR=\frac{CI}{RI\;},$$

where RI is the random consistency index.

The random index RI is proportional to the size of the (n×n) matrix. When the number of criteria *n* is less than 3, the consistency index—rather than the consistency ratio—is applied. It should be noted that the CR must not exceed 0.1; a *CR* beyond 0.1 indicates that the findings are inconsistent, and the assessment procedure must be reconsidered or repeated. Table 5 shows the *RI* for computing the *CR*.

### 5.2. Fuzzy TOPSIS

Following the completion of the AHP process to obtain the weight of each barrier and sub-barrier, the Fuzzy TOPSIS model is then used to rank the various WtE technologies for implementation in Ghana. The Technique for Order Performance by Similarity to Ideal Solution (TOPSIS) was proposed by Hwang and Yoon [71], and it is one of the most well-known techniques for solving MCDM problems. TOPSIS has been identified as an excellent multi-criteria decision-making technique due to its simplicity and ease of understanding. This method is based on the concept that the chosen alternative should have the shortest distance to the Positive Ideal Solution (PIS) (the solution which minimizes the cost criteria and maximizes the benefit criteria) and the farthest distance to the Negative Ideal Solution (NIS). The TOPSIS approach has many limitations in real-world applications, as it fails to give clear information on the situation and has ambiguous and undefined problems [72]. Therefore, Chen [73] extended TOPSIS with triangular FNs. He introduced a vertex method to calculate the distance between two triangular FNs. If $\tilde{x}$ = ($a_{1}$, $b_{1}$, $c_{1}$), $\tilde{y\;}\;$= ($a_{2}$, $b_{2}$, $c_{2}$) are two triangular FNs, then

(3) $$d\left(\tilde{x},\tilde{y}\right)∶=\sqrt{\frac{1}{3}\left[{\left(a_{1}-a_{2}\right)}^{2}+{\left(b_{1}-b_{2}\right)}^{2}+{\left(c_{1}-c_{2}\right)}^{2}\right]},$$

**Figure 5:**
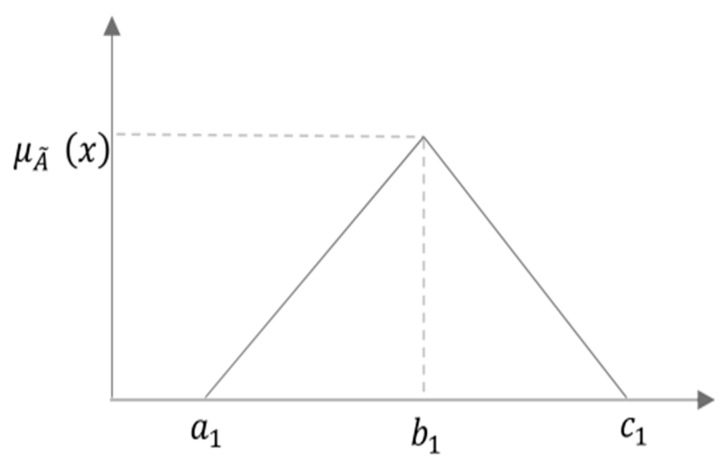
Triangular fuzzy number [78].

Regarding fuzzy set theory, as mentioned earlier, subjective human judgements are prone to uncertainties, ambiguity, and vagueness during the decision-making process. For the exactness of a selection, words can be more imprecise than numbers. As a result, Zadeh [74] developed the fuzzy set theory to assist decision-makers in reducing subjectivity and ambiguity [75,76]. A fuzzy set refers to a group of objects with a continuity of grades. Because of its simplicity, a triangular fuzzy number was used in this investigation. In order to better grasp and depict the qualitative qualities, a nine (0–9)-point hedonic scale is utilized (Table 6). Figure 5 also depicts the fuzzy numbers’ triangle membership function.

A fuzzy set is a group of objects with membership grades ranging from 0 to 1, with the membership grade being an intermediate value between 0 and 1. A fuzzy subset $\tilde{A}$ of a universal set, X is defined by a membership function $\mu \tilde{A}\left(x\right)\;$ which maps each element x in X to a real number [0, 1]. When an element’s grade of membership is 1, it implies that the element is absolutely included in that set. When the membership grade is 0, it indicates that the element is absolutely not part of that set. Values between 0 and 1 are allocated to ambiguous circumstances. A triangular fuzzy number can be shown as (a1, b1, c1). The parameters a1, b1 and c1 respectively denote the smallest possible value, the most promising value, and the largest possible value that describe a fuzzy event. In the following, some important definitions and notations of fuzzy set theory will be reviewed. The membership function of the triangular fuzzy numbers is shown in (2).

(4) $$\mu \tilde{A}\left(x\right)=\{\begin{array}{c} 0,\;\;\;\;\;\;\;\;\;\;\;\;x\langle a1\;or\;x\rangle c1\\ \frac{x-a1}{b1-a1},\;\;\;a1\leq x\leq b1\\ \frac{c1-x}{c1-b1},\;\;\;b1\leq x\leq c1 \end{array},$$

The fuzzy TOPSIS approach is detailed below; it was derived from Nădăban et al. [79]

Step 1: Assign a rating to the alternatives. We assume that we have a decision group with *K* members. The fuzzy rating of the *k^th^* decision maker regarding alternative $A_{i}$ with respect to criterion $C_{j}$ is denoted$\;{\tilde{x}}_{ij}^{k}=\left(a_{ij}^{k},\;b_{ij}^{k},c_{ij}^{k}\right)$.

Step 2: Compute the aggregated fuzzy ratings for the alternatives and the aggregated fuzzy weights for the criteria. The aggregated fuzzy rating ${\tilde{x}}_{ij}=\left(a_{ij},\;b_{ij},c_{ij}\right)\;$of the $i^{th}$ alternative w.r.t. $j^{th}$ criterion is obtained as follows:

(5) $$a_{ij}=\underset{k}{\min}\left\{a_{ij}^{k}\right\},\;b_{ij}=\frac{1}{K}\sum_{k=1}^{K}b_{ij}^{k},\;c_{ij}=\underset{k}{\max}\left\{c_{ij}^{k}\right\},$$

Step 3: Compute the normalized fuzzy decision matrix. The normalized fuzzy decision matrix is $\tilde{R}\;$= [${\tilde{r}}_{ij}$], where

(6) $${\tilde{r}}_{ij}=\left(\frac{a_{ij}}{c_{j}^{*}},\;\frac{b_{ij}}{c_{j}^{*}},\;\frac{c_{ij}}{c_{j}^{*}}\right)\;and\;c_{j}^{*}=\underset{i}{\max}\left\{c_{ij}\right\}\;\left(benefit\;criteria\right),$$

or

(7) $${\tilde{r}}_{ij}=\left(\frac{a_{j}^{-}}{c_{ij}},\;\frac{a_{j}^{-}}{b_{ij}},\;\frac{a_{j}^{-}}{a_{ij}}\right)\;and\;a_{j}^{-}=\underset{i}{\min}\left\{a_{ij}\right\}\;\left(non-benefit\;criteria\right),$$

Step 4: Compute the weighted normalized fuzzy decision matrix. The weighted normalized fuzzy decision matrix is $\tilde{V}\;$= (${\tilde{v}}_{ij}$), where ${\tilde{v}}_{ij}$ = ${\tilde{r}}_{ij}$ × $w_{j}$; here, the criteria weights $w_{j}$ are obtained from the AHP results

Step 5: Compute the Fuzzy Positive Ideal Solution (FPIS) and Fuzzy Negative Ideal Solution (FNIS). The FPIS and FNIS are calculated as follows:

(8) $$A^{*}=\left({\tilde{v}}_{1}^{*},{\tilde{v}}_{2}^{*},\ldots ,\;{\tilde{v}}_{n}^{*}\right),\;where\;{\tilde{v}}_{j}^{*}=\underset{i}{\max}\left\{v_{ij3}\right\},$$

and

(9) $$A^{-}=\left({\tilde{v}}_{1}^{-},{\tilde{v}}_{2}^{-},\ldots ,\;{\tilde{v}}_{n}^{-}\right),\;where\;{\tilde{v}}_{j}^{-}=\underset{i}{\min}\left\{v_{ij1}\right\},$$

Step 6: Compute the distance from each alternative to the FPIS and the FNIS. Let

(10) $$d_{i}^{*}=\overset{n}{\underset{j=1}{\sum }}d\left({\tilde{v}}_{ij},{\tilde{v}}_{j}^{*}\right),d_{i}^{-}=\overset{n}{\underset{j=1}{\sum }}d\left({\tilde{v}}_{ij},{\tilde{v}}_{j}^{-}\right)$$

be the distance from each alternative $A_{i}$ to the FPIS and to the FNIS, respectively.

Step 7: Compute the closeness coefficient $CC_{i}$ for each alternative. For each alternative $A_{i}$, we calculate the closeness coefficient $CC_{i}$ as follows:

(11) $$CC_{i\;}=\frac{d_{i}^{-}}{d_{i}^{-}+d_{i}^{*}},$$

Step 8: Prioritize the alternatives. The ideal scenario is the one that has the highest closeness coefficient.

### 5.3. Expert Survey Component

When deciding on a complicated problem, it is critical to appoint qualified experts, as these specialized professionals know how to manage and address the decision problem. Furthermore, experts’ opinions are crucial when major choices must be made quickly and data in a particular field are limited or unavailable. The goal of the survey in this study was to obtain expert opinion on various thermochemical and biochemical technologies, focusing on the most significant criteria, and to evaluate these technologies as waste management strategies as well as energy generation alternatives. Experts were carefully selected from institutions that are relevant to this field. Two academic experts were selected from the University of Energy and Natural Resources (Ghana), two from Tianjin University (China), and one from the University of KwaZulu-Natal (South Africa); one technical expert was selected from the Environmental Protection Agency (Ghana), two from the Energy Commission (Ghana), and two from the Ministry of Sanitation (Ghana). The procedures listed below include the qualitative research design for the expert survey:

1. The relevant criteria were selected for the assessment of WtE alternatives through the literature review.
2. A digital questionnaire was developed, and respondents were requested to access it online and fill in the required scores for each criterion depending on their relative importance in the decision-making process in order to assess various WtE alternatives based on the Saaty’s relative measurement scale.
3. The respondents were also required to rate how each criterion affects the alternatives using the fuzzy linguistic terms in order to rank the WtE technologies based on their feasibility against all of the criteria.

## 6. The Results and Discussion

A case is presented for the assessment of the technical, economic, environmental, and social feasibility of four WtE technologies—namely anaerobic digestion, conventional gasification, plasma arc gasification, and pyrolysis—in order to fully understand the practicability and efficiency of the suggested technology for investment and implementation in Ghana. The goal of this study is achieved by applying a multi-criteria model to select the optimal technology that is best suited for the Ghanaian context against the chosen evaluation criteria. The first part of this section presents the weights of the evaluation criteria and sub-criteria in terms of their significance for the advancement of the WtE technologies based on the judgements of the selected experts. Then, using this relationship (weight) in the establishment of the rankings for the various WtE alternatives, the experts scored the value of each WtE technology in the second part. The results of the sensitivity analysis are presented at the end of the section.

### 6.1. Criteria Weight Determination by AHP

The main evaluation criteria and sub-criteria results were acquired using the AHP weighting technique in this section. The following sub-sections show the aggregated priority vectors of the four main criteria and their respective sub-criteria derived from these weights.

#### 6.1.1. AHP Results of the Main Criteria

**Figure 6:**
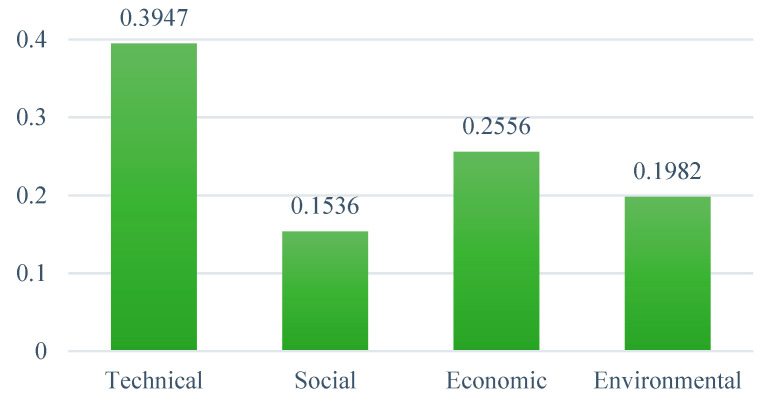
Priority weights of the main criteria.

The priority weights of the four main evaluation criteria are shown in Figure 6. The results indicate that the most significant criteria to influence the implementation of WtE technologies in Ghana are the technical criteria, with the highest weight of 0.395. This is followed by economic, environmental, and social criteria, with priority weights of 0.256, 0.198, and 0.154, respectively. These findings imply that WtE development in Ghana should prioritize assessing whether the selected technology is mature and reliable, as well as whether electricity generation and waste conversion are efficient enough. In order to achieve the goal of increasing the share of renewable energy in Ghana’s overall energy mix while efficiently treating MSW, it is vital to assess the feasibility of all of the technical factors. Cost is the next most important consideration for decision-makers when selecting a WtE technology. As a developing country with relatively lower economic growth, financial problems could be a crucial hindrance to WtE development in Ghana; therefore, selecting a cost-effective technology is considered very vital in achieving the main goal. Furthermore, the cost and technology are closely related, such that the cost reduces as the WtE technology improves. Because of the development in environmental consciousness in recent years, the environmental criterion is also becoming increasingly relevant. Unsurprisingly, this was reflected in the experts’ judgement, as MSW in Ghana is characterized by conventional and inefficient treatment methods that pose a serious threat to the environment and public health. Apart from the main benefits of energy generation and waste management, a WtE technology must be capable of reducing environmental impacts such as GHG emissions, soil, water and air pollution, and their related health impacts. The social aspect is generally the last thing to consider in WtE development in Ghana. The results of previous investigations on selecting sustainable energy sources and waste management strategies using either AHP subjective weights or other weighting methods are similar to the priority ranking of the main criteria established in this study [39,80,81].

#### 6.1.2. Technical Sub-Criteria

**Figure 7:**
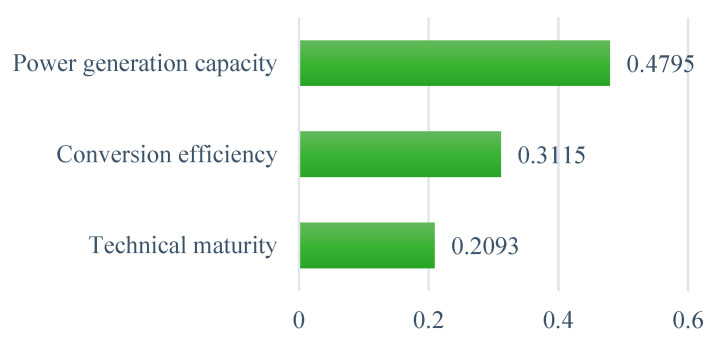
Priority weights of technical sub-criteria.

From the technical standpoint, the results in Figure 7 show that the power generation capacity is the most important sub-criterion to affect the selection of WtE technologies in Ghana, with the highest priority weight of 0.479. This is followed by conversion efficiency, with a weight of 0.312, and technical maturity, with a weight of 0.209. This means that regardless of the maturity or complexity of the technology adopted, the quantity of power generated by the WtE technology is the most important factor to consider when choosing it. Many similar case studies in other countries such as Nigeria [35], Russia [41], and Oman [33] also emphasized the relative significance of the power generation capacity of a WtE technology over other technological factors like conversion efficiency and technology maturity in the decision-making of the optimal selection.

#### 6.1.3. Economic Sub-Criteria

**Figure 8:**
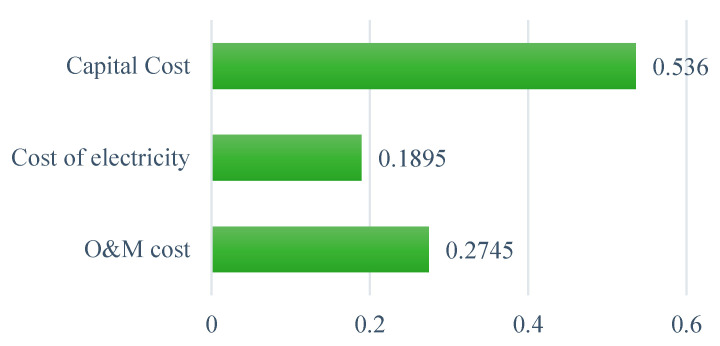
Priority weights of the economic sub-criteria.

Within the economic criteria, it can be seen in Figure 8 that the capital cost carries the highest importance in the selection of a WtE technology in Ghana, with an obtained weight of 0.536 and a large margin between this sub-criterion and the next significant sub-criterion. O&M cost is the next important sub-criterion, with a weight of 0.275, followed by the cost of electricity, with a weight of 0.189. Based on the experts’ judgements, the initial investment cost involved in the complete construction of a WtE facility in Ghana is more relevant than the technology’s O&M costs and the cost-per-unit generation of electricity. A similar observation was reported by previous studies in which the capital cost of WtE technologies gained the highest priority in decision-making concerning economic criteria [41,82].

#### 6.1.4. Environmental Sub-Criteria

**Figure 9:**
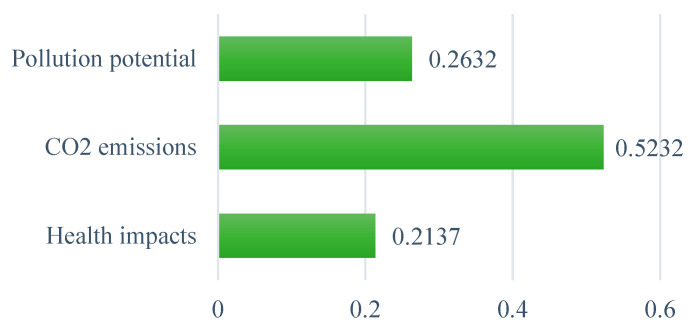
Priority weights of the environmental sub-criteria.

As shown in Figure 9, CO_2_ emissions are ranked as the most significant environmental sub-criterion to influence the decision of WtE selection in Ghana, with a priority weight of 0.523, followed by pollution potential of WtE technologies, with a weight of 0.263, and health impacts, with a weight of 0.214. Due to the increasing global concern about climate change impacts, many countries are making considerable efforts to reduce their greenhouse gas emissions, and Ghana is no exception. This concern is reflected in the experts’ opinion for the selections of an optimal WtE technology, such that priority should be given to a technology that can reduce CO_2_ emissions significantly. The wide gap between the weight of this criterion and the other environmental criteria emphasizes its importance in the development of WtE technologies in Ghana.

#### 6.1.5. Social Sub-Criteria

**Figure 10:**
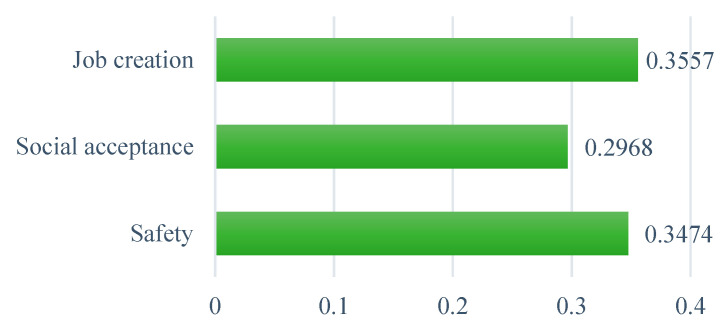
Priority weights of the social sub-criteria.

From the social perspective, the results in Figure 10 reveal that job creation is the most important sub-criterion to consider when selecting an optimal WtE technology for implementation in Ghana, with a priority weight of 0.358. The next important criterion in this context is safety, with a weight of 0.347, while the least important is societal acceptance, with a weight of 0.297. Because of the high unemployment rate in Ghana, greater focus is placed on the creation of new job possibilities among the social criteria. The relatively small gap between job creation and the safety criterion also indicates that the dangers and risks associated with constructing and operating a WtE facility are given almost equal attention when considering an optimal WtE technology selection in Ghana.

#### 6.1.6. The Overall Ranking of the Sub-Criteria

**Figure 11:**
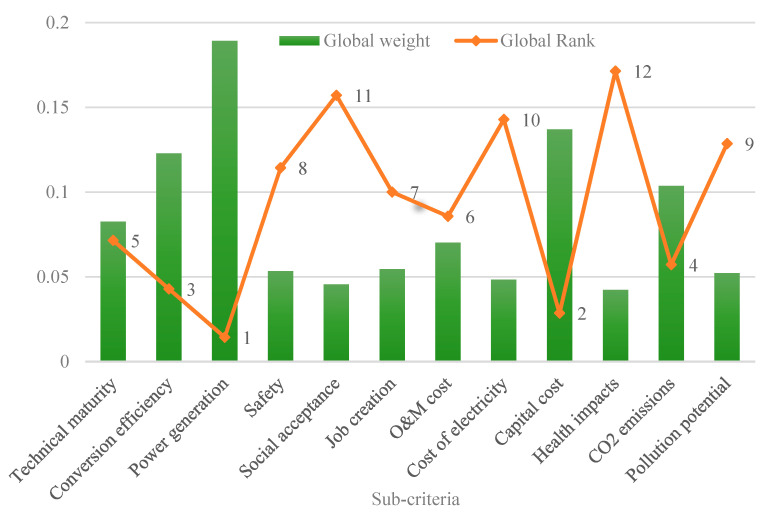
Overall priority weights and ranks of the sub-criteria.

Figure 11 shows the final priority weights of all of the sub-criteria evaluated in this study to select the optimal WtE technology for investment in Ghana. It also ranks the most important criterion to the least important when considering an optimal WtE selection. This section is different from the previous sections because it presents the overall rankings of all of the sub-criteria dependent on the weights of the respective main criteria. The aggregate weightings of the sub-criteria show that the power generation capacity has the highest priority in deciding the most attractive WtE technology in Ghana, with a final weight of 0.189, followed by capital cost, with a weight of 0.137, and conversion efficiency, with a weight of 0.123. On the other hand, the three least important criteria to influence the decision process are the cost of electricity, with a final weight of 0.048, social acceptance, with a weight of 0.045, and health impacts, with a weight of 0.042. This implies that for the WtE system to be viable in Ghana in terms of all of the specified parameters, the plant’s power generating capacity must be prioritized, while the health impact of said plant receives the least consideration. Intuitively, one could believe that human health is more valuable to most people than other criteria. However, the outcome does not always suggest that this is the case. It is possible that other criteria, such as CO_2_ emissions, are more closely linked to the evaluation of WtE technologies than health effects, and hence are given higher priority. Table 7 presents a summary of the local/global weights and ranks of the main criteria and sub-criteria for the selection of the optimal WtE technology in Ghana.

### 6.2. Ranking of the Alternatives by Fuzzy TOPSIS

**Figure 12:**
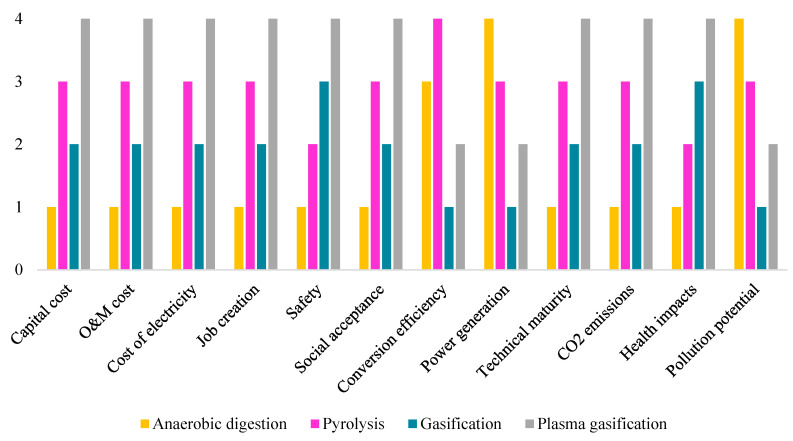
Ranks of the WtE technologies with regard to the evaluation criteria.

The findings from the Fuzzy TOPSIS theory application to choose the optimal WtE alternative in Ghana based on the four key sustainability criteria are presented in this section. The weighted normalized fuzzy decision matrix is computed based on the sub-criteria weights obtained from the previous section and shown in Table 8. The results for the fuzzy positive ideal solution (FPIS) and fuzzy negative ideal solution (FNIS) are presented in Table 9. The distances ($D^{+}$ and $D^{-}$) of each alternative from the positive and negative ideal solutions, $A^{+}$ and $A^{-}$, as well as the closeness coefficient $CC_{i}$, which were used to rank the different alternatives, are shown in Table 10 and Table 11, respectively. Figure 12 also shows the ranks of the WtE alternatives with regard to the evaluation criteria.

With a closeness coefficient of 0.63 to the ideal solution, anaerobic digestion ranks first as the optimal WtE alternative for the generation of electricity from MSW in Ghana, followed closely by gasification, with 0.56, pyrolysis, with 0.31, and plasma arc gasification as the least feasible WtE alternative, with 0.28. Anaerobic digestion was found to be the most appropriate for the overall aim; it also ranked first under economic, environmental, and social criteria, as shown in Figure 12. Anaerobic digestion offers superior economic performance over the remaining alternatives, with a reduced initial investment and operating costs. Moreover, it may result in energy recovery through biogas production, which contains 55–60% methane and can be utilized as fuel to generate electricity, resulting in cost savings to offset the capital and operating costs [83]. From the environmental perspective, apart from allowing the recycling of MSW by recovering renewable energy, anaerobic digestion also offers the potential to contribute to climate change mitigation by absorbing methane that would otherwise be emitted into the atmosphere. Given their direct relationship with climate change and global warming, CO_2_ emissions (kgCO_2_eq) obtained relatively high importance in the ecological assessment of the alternatives under consideration. Even though all of the selected WtE alternatives present lower CO_2_ emissions than Ghana’s current waste management strategies, anaerobic digestion was found to have the least damaging potential in terms of global warming [31,84]. Furthermore, Ghana has extensive experience with anaerobic digestion processes, as it is the most widely utilized WtE technology for the treatment of organic waste. Many operational small-scale anaerobic digesters in Ghana are mainly used for lighting and cooking. Due to its familiarity, anaerobic digestion has the highest level of social acceptance in Ghana. It also has the highest potential to create jobs for the Ghanaian residents, thereby reducing the unemployment rate in the country significantly. Even though anaerobic digestion’s development in Ghana is still in its initial stages, especially for large-scale plants, several policy frameworks and investments have been set aside to boost the progress of this technology for the generation of electricity from waste. Moreover, in recent years, several studies have focused on anaerobic digestion in Ghana to determine its feasibility and design policies for its incorporation into the country’s energy mix [85,86,87]. The greatest challenge to the development of anaerobic digestion for recovering energy from MSW in Ghana is the lack of waste separation. Because MSW in Ghana has varied components that are not sorted at the source, anaerobic digestion may be limited in energy production as the amount of producer gas needed for power production would be very low or negligible, making the whole system inefficient. This contributed to the poor performance of this WtE alternative under two of the most important evaluation criteria, i.e., power generation capacity and conversion efficiency. Therefore, efforts to support the development of anaerobic digestion in Ghana must include policies to enforce the source separation of waste in order to achieve the maximum benefit of this WtE technology.

Gasification was revealed to be the most favourable technology in terms of its power generation capacity and conversion efficiency, which are the first and third most important among all of the evaluation criteria. Despite this, gasification was ranked as the second most feasible WtE alternative in Ghana in terms of the overall goal. This could be attributed to gasification obtaining the first rank in only three out of twelve evaluation criteria. Nevertheless, this WtE alternative performed best among all of the thermochemical options in terms of all of the evaluation criteria except for health impacts and safety. Currently, the government of Ghana is concentrating its efforts on developing a plan to avoid the spread of uncontrolled dumpsites, as the current waste management system is failing to keep up with expanding waste volumes and requires significant waste reduction. Because gasification can produce a 50–90% decrease in waste volume [46], this technology is considered an appealing choice in this context, causing a significant reduction in land required for MSW disposal in Ghana. Moreover, this technology could be a viable solution to Ghana’s energy problem, with its net energy generation potential estimated to be between 20 and 26 kW/ton of MSW [88]. Furthermore, as high temperatures and abundant sunshine characterize Ghana’s climate, MSW may be thermally treated and fed into gasifiers with decreased moisture content. However, the relatively high capital and operating cost makes this alternative less feasible for a developing economy like Ghana. Tar cleaning in gasification also imposes an additional cost for this alternative, in contrast to the other alternatives. Waste segregation could also be a critical barrier to the development of gasification in Ghana, as the feedstock in this process immensely affects its efficiency and the chemical composition of the final products [88]. The electricity production from gasification is more significant when the wastes are combustible, such as paper, yard trimmings, wood, etc. Therefore, efforts towards effective waste segregation should be intensified in order to convert MSW into valuable products efficiently. Considering the technology maturity of gasification in Ghana, reports indicate that very few MSW gasification projects have been carried out on a pilot basis. Nonetheless, it is rated as the most socially acceptable technology among the thermochemical alternatives.

From a different perspective, plasma gasification could be the most appropriate method for MSW treatment in Ghana, where waste segregation is not performed effectively. This is because the plasma torch used in this process can break down any type of waste into its fundamental constituents without discrimination. Unfortunately, this technology is very limited in terms of its high capital and operating cost, as well as its high electricity demand. Even though the high conversion efficiency and electricity generation capacity make it attractive for an energy-poor country like Ghana, the construction of a plasma gasification plant is extremely costly, presumably due to its high demand for a higher level of plant automation, special construction materials to withstand the extreme temperatures, and the cost of plasma sources [89]. The extremely energy-intensive process of this technology also involves a substantial quantity of costly direct current power supply, which adds to the process’s operating cost and increases the capacity burden on the power grid. Consequently, energy-intensive processes like plasma gasification account for a significant portion of greenhouse gas emissions (about 30%) [89]. Technically, there is also a scarcity of experts in the field due to the significantly low maturity of this technology in a developing country like Ghana. The poor knowledge and understanding of this technology also create safety concerns for society and end-users due to its severe conditions for the processing of MSW.

The final WtE alternative ranking results of this study are comparable to previous studies in this field conducted for other developing countries or regions. Qazi et al. [33] compared eight biochemical and thermochemical WtE technologies for treating MSW in Oman using the AHP model for criteria weighting and alternative ranking. Their results also revealed anaerobic digestion as the most favourable technological option based on various technical, economic, environmental, and social criteria. However, the ranking results for the remaining technologies are inconsistent with the current work; in their work, plasma gasification obtained the second rank, followed by pyrolysis, with gasification being the least-preferred technology. This difference could be attributed to a number of reasons, which include geographical variations affecting waste quantity and composition, the different sub-criteria used for the evaluation, and the AHP method used for alternative ranking, which in the current work was replaced with the fuzzy TOPSIS method. The fuzzy set theory used in this study has the advantage of allowing decision-makers to use linguistic variables (TFNs) to minimize vagueness and ambiguity in human judgements. In another study, Wang et al. [36] prioritized four MSW treatment technologies for energy recovery based on the interval-valued fuzzy set theory. Their results also ranked anaerobic digestion as the most suitable MSW treatment scenario over incineration, landfill gas recovery, and gasification. Using objective weighting methods with data inclusion, Alao et al. [35] compared four WtE alternatives, namely anaerobic digestion, incineration, pyrolysis, and landfill gas recovery, against six sustainability sub-criteria under technical, economic, and environmental criteria to select the optimal technology for energy generation in Nigeria. This study used the Shannon entropy weighting method to obtain criteria weights, and the TOPSIS method for the ranking of alternatives. According to their results, anaerobic digestion ranked as the most preferred technology for energy generation in Nigeria, followed by pyrolysis, landfill gas recovery and incineration as the least preferred alternatives for standalone systems. This study concluded that the hybrid of anaerobic digestion, landfill gas recovery, and pyrolysis produces the best results in terms of environmental advantages and energy production potential. A similar approach was used by the same group of authors to prioritize WtE technologies in South Africa [90]. The overall results of this work also indicated that anaerobic digestion is the most feasible technology for energy generation from MSW in South Africa, while incineration ranked as the worst technological option.

### 6.3. Sensitivity Analysis

Sensitivity analysis shows how particular elements have a positive or negative effect on the outcome of decision-making. Because the criterion weight greatly influences the rank, any changes in the weight value should be carefully considered. By varying the initial weights of the various criteria, what will be the effect? A sensitivity analysis was used to determine the influence of underlying uncertainty in the experts’ judgments. The AHP results from the previous section indicated that technical criteria have the highest significance in the determination of the optimal WtE alternative and are thus considered the most sensitive. Therefore, in the current study, the global priority weights of the three technical criteria (power generation capacity, conversion efficiency, and technical maturity) were modified from 0.05 to 0.3 in increments of 0.05 in order to evaluate the influence of these key criteria on the overall ranking order of the alternatives.

**Figure 13:**
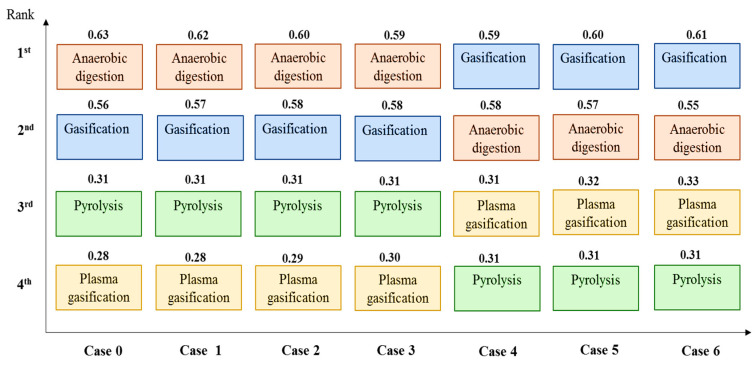
Sensitivity results of the criterion weight change on the ranking order for the WtE alternatives.

The results of the sensitivity analysis of the criteria weights are presented in this section. Table 12 shows the initial and modified criteria weights after increasing the technical criteria weights. Case 0 presents the initial weights obtained from the AHP results indicated in bold text. The weights of the three technical criteria, which were increased by 5% to 30% in steps of 5%, are also indicated in bold text. In order to maintain the total criteria weight at 1, the remaining criteria were proportionately adjusted. The revised criteria weights in Table 12 were applied to the fuzzy TOPSIS method to obtain new $CC_{i}$ values and, consequently, the new ranking order, as illustrated in Figure 13.

It can be seen that the ranking order from the first to the third scenario was maintained even though the $CC_{i}$ values changed slightly. Anaerobic digestion still ranked as the optimal WtE alternative even after the weights of the three technical criteria were increased by 5%, 10%, and 15%. The remaining alternatives also maintained their ranking orders after the first three scenarios, with gasification as the second most feasible technology, followed by pyrolysis and plasma gasification. This indicates a high level of robustness and consistency in the initial rankings. However, after the initial weights were increased by 20%, 25%, and 30% in scenarios 4 to 6, the ranking order of all of the WtE alternatives changed alongside the $CC_{i}\;$values. Gasification became the first technology, followed by anaerobic digestion, plasma gasification, and pyrolysis, which ranked as the least preferred technology. This could be attributed to the fact that more emphasis was placed on the technical criteria, which carry the highest weight among all of the evaluation criteria. Therefore, the results favour gasification and plasma gasification, as they performed best in the two most important technical criteria, i.e., power generation capacity and conversion efficiency. This implies that when decision-makers and stakeholders focus on the ultimate goal of selecting a technology that can adequately generate electricity for Ghana while ignoring other factors, gasification would be the best alternative, followed by plasma gasification. The energy conversion process of anaerobic digestion, on the other hand, is not as efficient as the thermochemical processes; therefore, if more priority is given to the technical factors, this alternative will be less favoured for selection in Ghana.

It is worth noting that even though the ranking order of pyrolysis changed after the third scenario, its $CC_{i}$ value was constant throughout all the different scenarios. This shows that this alternative is not sensitive to the technical criteria for this study. Pyrolysis ranked third for all three technical criteria, indicating that it is neither the best nor worst under these criteria. Therefore, changing the criteria weights does not significantly impact the overall priority value. Pyrolysis ranked as the least preferred technology in scenarios 4–6 because, despite the high costs involved in its establishment, the energy yield from this technology is also relatively low (only second to anaerobic digestion).

The sensitivity analysis results suggest that the combined decision framework is sensitive to the technical criteria. As a result, they must be given serious attention during the pragmatic decision-making process by identifying the design and requirements of the country’s power generation industry ahead of time. An integrated system combining gasification and AD technologies, rather than their standalones, might be ideal for the achievement of a well-balanced performance across various criteria. Similar studies conducted in the past have suggested that a waste-to-energy hybrid system that combines anaerobic digestion and gasification is not only cost-effective but also offers the greatest potential for recovering energy from waste [90,91].

## 7. Conclusions

The current study applied an integrated multi-criteria decision-making method to the optimal selection of waste-to-energy technologies for implementation in Ghana. The analytical hierarchy process, which allows a decision-maker the flexibility to quantitatively rank criteria with respect to the overall goal using priority calculations, was applied to determine the weights of the evaluation criteria. Consequently, the fuzzy TOPSIS method, which helps to reduce ambiguity and vagueness in human judgement using linguistic variables, was used to rank WtE alternatives. In this study, four WtE technologies (anaerobic digestion, gasification, pyrolysis, and plasma gasification) were evaluated against twelve indicators covering technical, economic, environmental, and social criteria.

The priority weighting results of the main criteria indicated that technical criteria are the most important, followed by economic and environmental criteria, while social criteria ranked as the least important in the selection process. Amongst the sub-criteria, the power generation capacity was given the highest consideration, while health impacts obtained the lowest weight. The overall ranking results of the WtE alternatives reveal that anaerobic digestion is the most suitable technology for adoption in the Ghanaian context, given the relative importance of the key criteria. Gasification and pyrolysis ranked in second and third place, respectively, while plasma gasification was considered the worst alternative. In order to determine the stability and robustness of the proposed model, a sensitivity analysis was carried out to evaluate the degree of reliability of the obtained results. The results demonstrated a high degree of consistency and robustness, irrespective of the variations in the baseline scenario. It was also deduced from the sensitivity analysis that if more emphasis is placed on the most critical criteria (i.e., technical), the optimal selection shifts towards gasification technology; therefore, a hybrid system combining both anaerobic digestion and gasification technologies might be more viable for energy generation and waste management in Ghana.

According to Ghana’s current renewable energy master plan, electricity generation from MSW is anticipated to reach 50.1 MW of utility-scale power in 2030 in order to contribute to the country’s overall energy supply mix while reducing the environmental consequences of energy production. The implementation of a hybrid of anaerobic digestion and gasification, both of which are suitable renewable energy production technologies, can help to achieve this goal. The municipal solid waste composition in Ghana is particularly suitable for energy recovery by these two technologies. The two technologies also have the benefit of producing little to no CO_2_ emissions, which can assist Ghana in accomplishing its goal of reducing GHG emissions. In addition to producing green electricity, these two technologies can have important urban, industrial, and transportation applications to improve the socioeconomic and environmental needs of the country. For instance, the production of biogas from anaerobic digestion may promote clean cooking and provide a solution to numerous socioeconomic and health issues. Liquid bio-carbon dioxide, a byproduct of converting biogas and syngas, may be stored and used as a chemical platform for various industrial purposes. In order to enhance green transportation, high-quality fuels may be generated, such as bio-methane (biogas from anaerobic digestion) and hydrogen gas (syngas from gasification). In order to realize the above-mentioned practical implications of the anaerobic digestion and gasification technologies, this paper recommends that the decision-makers and stakeholders adopt the following measures: (1) create avenues for foreign investment and establish particular financial assistance for industries that use both technologies for MSW treatment, such as low interest and subsidies; (2) make conscious efforts to set realistic targets that increases the share of WtE technologies in Ghana’s total installed renewable generation capacity; (3) train more engineers in these two technologies to enhance the skill set; and (4) encourage proper waste segregation among the public in order to improve the efficiency of both anaerobic digestion and gasification.

It is worth noting that the current work has its share of limitations. First, the proposed model relies solely on experts’ judgement to rank various WtE alternatives, thereby lacking a certain degree of objectivity, which could be supplemented by using actual data with units to express the alternatives in relation to the selection criteria. Thus, future studies should develop a method for the integration of experts’ judgement with the input data of various alternatives in order to obtain an optimal selection so that the benefits of both subjective and objective techniques may be realized. Next, the twelve selection criteria—spanning only technical, economic, environmental, and social factors—taken into account in this study appear not to be all-inclusive. In order to guarantee that the evaluation procedure is thorough, future studies must consider additional selection criteria, including those related to political, regulatory, and institutional capacity. These are needed in order to emphasize the concept of a circular economy in waste management and the production of renewable energy. Furthermore, with the development of new and better technologies in the global WtE market, research in the future can assess the feasibility of new and growing alternatives for integration into the Ghanaian system. In the future, a specialized study will be required in order to assess the conditions under which WtE projects and infrastructure may be modelled to fit the country’s specific needs.

## Figures and Tables

**Table 1:** REMP targets for WtE technologies in Ghana [19].

Technology/Source		Units	Reference (2015)	2020	2025	2030
Utility scale power	MSW	MW	0.1	0.1	30.1	50.1
Biogas	Agricultural/Industrial organic waste	Units	10	30	100	200
	Institutional	Units	<100	180	320	500
	Domestic		<50	80	130	200

**Table 3:** Summary of the evaluation criteria.

Main Criteria	Sub-Criteria (Unit)	Description	Criteria Factor
Technical	Conversion efficiency	This is among the major aspects of energy systems. This metric is calculated by dividing the usable output by the entire input.	Beneficial
	Power generation capacity (kW/tMSW)	Under specific conditions, this is the highest amount of energy that a WtE system can potentially produce.	Beneficial
	Technology maturity	This criterion denotes the stage of development of the WtE plant, i.e., whether it is in the experimental or commercial usage stages. Commercial technology is mostly preferable.	Beneficial
Economic	Capital cost (US$)	This is the initial investment required to build a WtE facility. Purchase of mechanical equipment, facility and device estimations, infrastructure costs, technological installations, preliminary funds, interest payments, and land use are all included.	Non-beneficial
	O&M cost (US$)	This includes the expenses of running a power plant, split into two groups. The first is the operating expenditure, which comprises staff wages along with expenditure on power, commodities, and structures for the functioning of the energy system. Another cost is maintenance, which extends the life of an energy equipment and prevents problems that might lead it to stop functioning. O&M expenses may be extremely expensive; therefore, it is considered more sustainable for a system to minimize these costs.	Non-beneficial
	Cost of energy (US$/kWh)	This involves the cost of generating one unit of energy. Employing technology that generates energy at the lowest possible cost is preferable.	Non-beneficial
Environmental	CO_2_ emissions (ktCO_2_eq)	For WtE technologies, carbon dioxide is released during plant operation as a result of the carbon content of MSW. The technology with lower carbon dioxide emissions is preferred.	Non-beneficial
	Health impacts	The ability of the chosen WtE technology to reduce hazards to public health and the health of the employees.	Non-beneficial
	Pollution potential	This refers to the harmful environmental effects of WtE technologies on water, soil, and air.	Non-beneficial
Social	Social acceptance	The term “social acceptability” refers to a broad range of local public opinion on energy systems. It is crucial because public opinion and pressure groups may have a big impact on how long it takes to finish a big energy project.	Beneficial
	Job creation	Prospects of job opportunities to be generated by the WtE project. In the decision-making process of local governments, job creation of energy systems is indispensably considered and selected to evaluate their contributions.	Beneficial
	Safety	The danger of fire, explosion, and health risks associated with constructing and operating a WtE plant are all taken into account.	Beneficial

**Table 4:** Saaty’s basic scale of absolute numbers [68].

Numerical Representations	Definition
1	Equally important
3	Slightly important
5	Strongly important
7	Very strongly important
9	Extremely important
2,4,6 and 8	Represent values in between
1/1, 1/3, 1/5, 1/7, 1/9	Represent reciprocal values

**Table 5:** Random consistency index values for computing the consistency ratio [70].

n	3	4	5	6	7	8	9	10
*RI*	0.5247	0.8816	1.1086	1.2479	1.3417	1.4057	1.4499	1.4854

**Table 6:** Linguistic variables of the project alternatives [77].

Linguistic Variable	Fuzzy Numbers
Worst (W)	(1,1,3)
Poor (P)	(1,3,5)
Fair (F)	(3,5,7)
Good (G)	(5,7,9)
Best (B)	(7,9,9)

**Table 7:** Summary of the weights and ranks of the evaluation criteria for WtE technology selection.

Main Criteria	Weight	Sub-Criteria	Local Weight	Local Rank	Global Weight	Global Rank
Technical	0.395	Technical maturity	0.209	3	0.083	5
		Conversion efficiency	0.316	2	0.123	3
		Power generation capacity	0.479	1	0.189	1
Social	0.154	Safety	0.347	2	0.053	8
		Social acceptance	0.297	3	0.045	11
		Job creation	0.356	1	0.054	7
Economic	0.256	O&M cost	0.275	2	0.070	6
		Cost of electricity	0.189	3	0.048	10
		Capital cost	0.536	1	0.137	2
Environmental	0.198	Health impacts	0.214	3	0.042	12
		CO_2_ emissions	0.523	1	0.104	4
		Pollution potential	0.263	2	0.052	9

**Table 8:** Weighted normalized fuzzy decision matrix.

	IN			OM			COE			JC		
AD	0.02	0.02	0.05	0.01	0.01	0.01	0.01	0.01	0.02	0.02	0.04	0.06
PYR	0.02	0.04	0.14	0.01	0.02	0.07	0.01	0.01	0.05	0.01	0.02	0.06
GAS	0.02	0.03	0.14	0.01	0.01	0.07	0.01	0.01	0.02	0.01	0.03	0.06
PAG	0.02	0.05	0.14	0.01	0.03	0.07	0.01	0.02	0.05	0.01	0.02	0.06
	SF			SA			CE			PGC		
AD	0.01	0.04	0.05	0.03	0.04	0.05	0.01	0.06	0.12	0.02	0.06	0.15
PYR	0.01	0.03	0.05	0.01	0.02	0.05	0.01	0.05	0.12	0.02	0.13	0.19
GAS	0.01	0.03	0.05	0.01	0.03	0.05	0.07	0.12	0.12	0.11	0.18	0.19
PAG	0.01	0.02	0.04	0.01	0.01	0.04	0.01	0.08	0.12	0.11	0.16	0.19
	TM			CO_2_			HI			PP		
AD	0.05	0.07	0.08	0.01	0.01	0.03	0.00	0.01	0.01	0.01	0.02	0.05
PYR	0.01	0.04	0.08	0.01	0.02	0.10	0.00	0.01	0.04	0.01	0.01	0.02
GAS	0.01	0.04	0.08	0.01	0.05	0.10	0.00	0.01	0.04	0.01	0.01	0.01
PAG	0.01	0.02	0.06	0.02	0.05	0.10	0.00	0.02	0.04	0.01	0.01	0.02

**Table 9:** Fuzzy Positive Ideal Solution (FPIS) and Fuzzy Negative Ideal Solution (FNIS).

	IN			OM			COE			JC		
$A^{*}$	0.015	0.019	0.046	0.008	0.008	0.014	0.005	0.006	0.016	0.018	0.044	0.055
$A^{-}$	0.020	0.053	0.137	0.008	0.025	0.070	0.005	0.016	0.048	0.006	0.017	0.055
	SF			SA			CE			PGC		
$A^{*}$	0.006	0.041	0.053	0.026	0.041	0.046	0.068	0.118	0.123	0.105	0.176	0.189
$A^{-}$	0.006	0.015	0.041	0.005	0.013	0.036	0.014	0.052	0.123	0.021	0.059	0.147
	TM			CO_2_			HI			PP		
$A^{*}$	0.046	0.074	0.083	0.012	0.013	0.035	0.005	0.006	0.014	0.006	0.007	0.010
$A^{-}$	0.009	0.017	0.065	0.021	0.047	0.104	0.005	0.019	0.042	0.007	0.024	0.052

**Table 10:** Distances between the WtE alternatives and $A^{*}$ , $A^{-}$ with regard to each criterion.

	IN	OM	COE	JC	SF	SA	CE	PGC	TM	CO_2_	HI	PP	D+
$DA^{*}$	0.00	0.00	0.00	0.00	0.00	0.00	0.05	0.09	0.00	0.00	0.00	0.03	0.16
	0.05	0.03	0.02	0.01	0.01	0.02	0.05	0.06	0.03	0.04	0.02	0.00	0.33
	0.05	0.03	0.00	0.01	0.01	0.01	0.00	0.00	0.03	0.04	0.02	0.00	0.20
	0.06	0.03	0.02	0.02	0.02	0.02	0.04	0.01	0.04	0.04	0.02	0.00	0.32
	IN	OM	COE	JC	SF	SA	CE	PGC	TM	CO_2_	HI	PP	D−
$DA^{-}$	0.06	0.03	0.02	0.02	0.02	0.02	0.00	0.00	0.04	0.04	0.02	0.00	0.27
	0.01	0.01	0.00	0.00	0.01	0.01	0.00	0.05	0.02	0.02	0.01	0.02	0.15
	0.01	0.01	0.02	0.01	0.01	0.01	0.05	0.09	0.02	0.00	0.00	0.03	0.26
	0.00	0.00	0.00	0.00	0.00	0.00	0.02	0.08	0.00	0.00	0.00	0.02	0.12

**Table 11:** Computations of $D^{+}$ , $D^{-}$ and $CC_{i}$

WtE Alternative	$D^{+}$	$D^{-}$	$D^{+}+D^{-}$	$CC_{i}$	Rank
Anaerobic digestion	0.16	0.27	0.43	0.63	1
Pyrolysis	0.33	0.15	0.48	0.31	3
Gasification	0.20	0.26	0.46	0.56	2
Plasma arc gasification	0.32	0.12	0.44	0.28	4

**Table 12:** Initial and modified criteria weights after increasing the technical criteria weights by 5% to 30%.

Sub-Criteria	Case 0 (Initial Weights)	Case 1 (5%)	Case 2 (10%)	Case 3 (15%)	Case 4 (20%)	Case 5 (25%)	Case 6 (30%)
Capital cost	0.137	0.135	0.132	0.130	0.128	0.126	0.124
O&M cost	0.070	0.068	0.065	0.063	0.061	0.059	0.057
Cost of electricity	0.048	0.046	0.043	0.041	0.039	0.037	0.035
Job creation	0.055	0.053	0.050	0.048	0.046	0.044	0.042
Safety	0.053	0.051	0.048	0.046	0.044	0.042	0.040
Social acceptance	0.046	0.044	0.041	0.039	0.037	0.035	0.033
Conversion efficiency	0.123	0.129	0.135	0.141	0.148	0.154	0.160
Power generation	0.189	0.198	0.208	0.217	0.227	0.236	0.246
Technical maturity	0.083	0.087	0.091	0.095	0.100	0.104	0.108
CO_2_ emissions	0.104	0.102	0.099	0.097	0.095	0.093	0.091
Health impacts	0.042	0.040	0.037	0.035	0.033	0.031	0.029
Pollution potential	0.052	0.050	0.047	0.045	0.043	0.041	0.039

## Data Availability

Not applicable.

## References

[1] BP (2020). Statistical Review of World Energy—All Data 1965–2019.

[2] Miao Z., Shastri Y., Grift T.E., Hansen A.C., Ting K. (2012). Lignocellulosic biomass feedstock transportation alternatives, logistics, equipment configurations, and modeling. Biofuels Bioprod. Biorefin..

[3] Panwar N.L., Kaushik S.C., Kothari S. (2011). Role of renewable energy sources in environmental protection: A review. Renew. Sustain. Energy Rev..

[4] Ayodele T., Alao M., Ogunjuyigbe A., Munda J. (2019). Electricity generation prospective of hydrogen derived from biogas using food waste in south-western Nigeria. Biomass Bioenergy.

[5] USEPA National Overview: Facts and Figures on Materials, Wastes and Recycling. https://www.epa.gov/facts-and-figures-about-materials-waste-and-recycling/forms/contact-us-about-facts-and-figures.

[6] Nixon J., Dey P., Ghosh S., Davies P. (2013). Evaluation of options for energy recovery from municipal solid waste in India using the hierarchical analytical network process. Energy.

[7] Trindade A.B., Palacio J.C.E., González A.M., Orozco D.J.R., Lora E.E.S., Renó M.L.G., del Olmo O.A. (2018). Advanced exergy analysis and environmental assesment of the steam cycle of an incineration system of municipal solid waste with energy recovery. Energy Convers. Manag..

[8] International Renewable Energy Agency (IRENA) Renewable Energy Statistics. http://www.irena.org/DocumentDownloads/Publications/IRENA_RE_Statistics_2016.pdf.

[9] Brunner P.H., Rechberger H. (2015). Waste to energy—Key element for sustainable waste management. Waste Manag..

[10] Joseph L.P., Prasad R. (2019). Assessing the sustainable municipal solid waste (MSW) to electricity generation potentials in selected Pacific Small Island Developing States (PSIDS). J. Clean. Prod..

[11] Yap H.Y., Nixon J.D. (2015). A multi-criteria analysis of options for energy recovery from municipal solid waste in India and the UK. Waste Manag..

[12] Geiger M.T., Trenczek J., Wacker K.M. (2019). Understanding economic growth in Ghana in comparative perspective. World Bank Policy Res. Work. Pap..

[13] Energy Commission Ghana (2020). National Energy Statistics 2000–2019.

[14] Energy Commission Ghana (2021). Energy (Demand and Supply) Outlook for Ghana.

[15] Gyamfi S., Diawuo F.A., Kumi E.N., Sika F., Modjinou M. (2018). The energy efficiency situation in Ghana. Renew. Sustain. Energy Rev..

[16] Statista Share of Population with Access to Electricity in Ghana from 2008 to 2019. https://www.statista.com/statistics/1118772/population-access-to-electricity-in-ghana/.

[17] Agyekum E.B. (2021). Techno-economic comparative analysis of solar photovoltaic power systems with and without storage systems in three different climatic regions, Ghana. Sustain. Energy Technol. Assess..

[18] Energy Commission Ghana (2018). Energy (Demand and Supply) Outlook for Ghana.

[19] Energy Commission (2019). Ghana Renewable Energy Master Plan.

[20] Miezah K., Obiri-Danso K., Kádár Z., Fei-Baffoe B., Mensah M.Y. (2015). Municipal solid waste characterization and quantification as a measure towards effective waste management in Ghana. Waste Manag..

[21] Ofori-Boateng C., Lee K.T., Mensah M. (2013). The prospects of electricity generation from municipal solid waste (MSW) in Ghana: A better waste management option. Fuel Process. Technol..

[22] Kemausuor F., Kamp A., Thomsen S.T., Bensah E., Østergård H. (2014). Assessment of biomass residue availability and bioenergy yields in Ghana. Resour. Conserv. Recycl..

[23] Amo-Asamoah E., Owusu-Manu D.-G., Asumadu G., Ghansah F.A., Edwards D.J. (2020). Potential for waste to energy generation of municipal solid waste (MSW) in the Kumasi metropolis of Ghana. Int. J. Energy Sect. Manag..

[24] Aslam F., Elkotb M.A., Iqtidar A., Khan M.A., Javed M.F., Usanova K.I., Khan M.I., Alamri S., Musarat M.A. (2022). Compressive strength prediction of rice husk ash using multiphysics genetic expression programming. Ain Shams Eng. J..

[25] Iqtidar A., Khan N.B., Kashif-Ur-Rehman S., Javed M.F., Aslam F., Alyousef R., Alabduljabbar H., Mosavi A. (2021). Prediction of Compressive Strength of Rice Husk Ash Concrete through Different Machine Learning Processes. Crystals.

[26] Asante D., Ampah J.D., Afrane S., Adjei-Darko P., Asante B., Fosu E., Dankwah D.A., Amoh P.O. (2022). Prioritizing strategies to eliminate barriers to renewable energy adoption and development in Ghana: A CRITIC-fuzzy TOPSIS approach. Renew. Energy.

[27] Agyekum E.B., Amjad F., Mohsin M., Ansah M.N.S. (2021). A bird’s eye view of Ghana’s renewable energy sector environment: A Multi-Criteria Decision-Making approach. Util. Policy.

[28] Wang J.-J., Jing Y.-Y., Zhang C.-F., Zhao J.-H. (2009). Review on multi-criteria decision analysis aid in sustainable energy decision-making. Renew. Sustain. Energy Rev..

[29] Bortoluzzi M., de Souza C.C., Furlan M. (2021). Bibliometric analysis of renewable energy types using key performance indicators and multicriteria decision models. Renew. Sustain. Energy Rev..

[30] Milutinović B., Stefanović G., Đekić P.S., Mijailović I., Tomić M. (2017). Environmental assessment of waste management scenarios with energy recovery using life cycle assessment and multi-criteria analysis. Energy.

[31] Fernández-González J., Grindlay A., Serrano-Bernardo F., Rodríguez-Rojas M., Zamorano M. (2017). Economic and environmental review of Waste-to-Energy systems for municipal solid waste management in medium and small municipalities. Waste Manag..

[32] Afrane S., Ampah J.D., Jin C., Liu H., Aboagye E.M. (2021). Techno-economic feasibility of waste-to-energy technologies for investment in Ghana: A multicriteria assessment based on fuzzy TOPSIS approach. J. Clean. Prod..

[33] Qazi W.A., Abushammala M.F., Azam M.-H. (2018). Multi-criteria decision analysis of waste-to-energy technologies for municipal solid waste management in Sultanate of Oman. Waste Manag. Res..

[34] Abdallah M., Shanableh A., Arab M., Shabib A., Adghim M., El-Sherbiny R. (2019). Waste to energy potential in middle income countries of MENA region based on multi-scenario analysis for Kafr El-Sheikh Governorate, Egypt. J. Environ. Manag..

[35] Alao M., Ayodele T., Ogunjuyigbe A., Popoola O. (2020). Multi-criteria decision based waste to energy technology selection using entropy-weighted TOPSIS technique: The case study of Lagos, Nigeria. Energy.

[36] Wang Z., Ren J., Goodsite M., Xu G. (2018). Waste-to-energy, municipal solid waste treatment, and best available technology: Comprehensive evaluation by an interval-valued fuzzy multi-criteria decision making method. J. Clean. Prod..

[37] Shah S.A.A., Longsheng C., Solangi Y.A., Ahmad M., Ali S. (2020). Energy trilemma based prioritization of waste-to-energy technologies: Implications for post-COVID-19 green economic recovery in Pakistan. J. Clean. Prod..

[38] Fetanat A., Mofid H., Mehrannia M., Shafipour G. (2019). Informing energy justice based decision-making framework for waste-to-energy technologies selection in sustainable waste management: A case of Iran. J. Clean. Prod..

[39] Islam S., Ponnambalam S.G., Lam H.L. (2016). A Sustainable Energy Planning Endeavor for Selecting a Waste-to-Energy Option. Proceedings of the 4th International Conference on the Development in the in Renewable Energy Technology (ICDRET).

[40] Alao M.A., Popoola O.M., Ayodele T.R. (2022). A novel fuzzy integrated MCDM model for optimal selection of waste-to-energy-based-distributed generation under uncertainty: A case of the City of Cape Town, South Africa. J. Clean. Prod..

[41] Kurbatova A., Abu-Qdais H. (2020). Using Multi-Criteria Decision Analysis to Select Waste to Energy Technology for a Mega City: The Case of Moscow. Sustainability.

[42] Osei-Appiah A., Dioha M. (2019). Techno-economic assessment of waste-to-energy technologies in Ghana. J. Sustain. Energy.

[43] Agbejule A., Shamsuzzoha A., Lotchi K., Rutledge K. (2021). Application of Multi-Criteria Decision-Making Process to Select Waste-to-Energy Technology in Developing Countries: The Case of Ghana. Sustainability.

[44] Zavadskas E.K., Govindan K., Antucheviciene J., Turskis Z. (2016). Hybrid multiple criteria decision-making methods: A review of applications for sustainability issues. Econ. Res. Ekon. Istraživanja.

[45] AlQattan N., Acheampong M., Jaward F.M., Ertem F.C., Vijayakumar N., Bello T. (2018). Reviewing the potential of Waste-to-Energy (WTE) technologies for Sustainable Development Goal (SDG) numbers seven and eleven. Renew. Energy Focus.

[46] Ouda O.K.M., Raza S.A., Nizami A.S., Rehan M., Al-Waked R., Korres N.E. (2016). Waste to energy potential: A case study of Saudi Arabia. Renew. Sustain. Energy Rev..

[47] Bosmans A., Vanderreydt I., Geysen D., Helsen L. (2012). The crucial role of Waste-to-Energy technologies in enhanced landfill mining: A technology review. J. Clean. Prod..

[48] Miranda M.L., Hale B. (1997). Waste not, want not: The private and social costs of waste-to-energy production. Energy Policy.

[49] Tan S.T., Ho W.S., Hashim H., Lee C.T., Taib M.R., Ho C.S. (2015). Energy, economic and environmental (3E) analysis of waste-to-energy (WTE) strategies for municipal solid waste (MSW) management in Malaysia. Energy Convers. Manag..

[50] Vogg H., Metzger M., Stieglitz L. (1987). Recent findings on the formation and decomposition of PCDD/PCDF in municipal solid waste incineration. Waste Manag. Res..

[51] Themelis N. (2003). An overview of the global waste-to-energy industry. Waste Manag. World.

[52] Jaeger M., Mayer M. (2000). The Noell Conversion Process—A gasification process for the pollutant-free disposal of sewage sludge and the recovery of energy and materials. Water Sci. Technol..

[53] Arena U. (2012). Process and technological aspects of municipal solid waste gasification. A review. Waste Manag..

[54] Young G. (2010). Municipal Solid Waste to Energy Conversion Processes: Economic, Technical, and Renewable Comparisons.

[55] Malkow T. (2004). Novel and innovative pyrolysis and gasification technologies for energy efficient and environmentally sound MSW disposal. Waste Manag..

[56] Young G. (2010). Introduction to Gasification/Pyrolysis and Combustion Technology(s). Municipal Solid Waste to Energy Conversion Processes: Economic, Technical, and Renewable Comparisons.

[57] Ducharme C. (2010). Technical and Economic Analysis of Plasma-Assisted Waste-to-Energy Processes. Master’s Thesis.

[58] Tavares J.R., Rao L., Derboghossian C., Carabin P., Kaldas A., Chevalier P., Holcroft G. (2011). Large-Scale Plasma Waste Gasification. IEEE Trans. Plasma Sci..

[59] Stolarek P., Ledakowicz S. (2001). Thermal processing of sewage sludge by drying, pyrolysis, gasification and combustion. Water Sci. Technol..

[60] Bridgwater A. (2003). Renewable fuels and chemicals by thermal processing of biomass. Chem. Eng. J..

[61] Katyal S. (2007). Effect of Carbonization Temperature on Combustion Reactivity of Bagasse Char. Energy Sources Part A Recovery Util. Environ. Eff..

[62] Mohan D., Pittman C.U., Steele P.H. (2006). Pyrolysis of Wood/Biomass for Bio-oil: A Critical Review. Energy Fuels.

[63] Sawatdeenarunat C., Surendra K., Takara D., Oechsner H., Khanal S.K. (2015). Anaerobic digestion of lignocellulosic biomass: Challenges and opportunities. Bioresour. Technol..

[64] Nguyen D., Gadhamshetty V., Nitayavardhana S., Khanal S.K. (2015). Automatic process control in anaerobic digestion technology: A critical review. Bioresour. Technol..

[65] Santi G., Proietti S., Moscatello S., Stefanoni W., Battistelli A. (2015). Anaerobic digestion of corn silage on a commercial scale: Differential utilization of its chemical constituents and characterization of the solid digestate. Biomass Bioenergy.

[66] Weitz K.A., Thorneloe S.A., Nishtala S.R., Yarkosky S., Zannes M. (2002). The Impact of Municipal Solid Waste Management on Greenhouse Gas Emissions in the United States. J. Air Waste Manag. Assoc..

[67] Saaty T.L. (1980). The Analytic Hierarchy Process: Planning, Priority Setting, Resource Allocation.

[68] Saaty T.L. (2008). Decision making with the analytic hierarchy process. Int. J. Serv. Sci..

[69] Awasthi A., Chauhan S.S. (2012). A hybrid approach integrating Affinity Diagram, AHP and fuzzy TOPSIS for sustainable city logistics planning. Appl. Math. Model..

[70] Brunelli M. (2015). Introduction to the Analytic Hierarchy Process.

[71] Hwang C.L., Yoon K.P. (1981). Multiple Attribute Decision Making. Methods and Applications. A State-of—The-Art Survey.

[72] Madi E.N., Garibaldi J.M., Wagner C. (2016). An exploration of issues and limitations in current methods of TOPSIS and fuzzy TOPSIS. Proceedings of the 2016 IEEE International Conference on Fuzzy Systems (FUZZ-IEEE).

[73] Chen C.-T. (2000). Extensions of the TOPSIS for group decision-making under fuzzy environment. Fuzzy Sets Syst..

[74] Zadeh L.A. (1978). Fuzzy sets as a basis for a theory of possibility. Fuzzy Sets Syst..

[75] Novák V. (2012). Reasoning about mathematical fuzzy logic and its future. Fuzzy Sets Syst..

[76] Roszkowska E., Kusterka-Jefmańska M., Jefmański B. (2021). Intuitionistic Fuzzy TOPSIS as a Method for Assessing Socioeconomic Phenomena on the Basis of Survey Data. Entropy.

[77] Wu Y., Xu C., Zhang T. (2018). Evaluation of renewable power sources using a fuzzy MCDM based on cumulative prospect theory: A case in China. Energy.

[78] Liu X., Chen Y. (2013). A Systematic Approach to OptimizinghhValue for Fuzzy Linear Regression with Symmetric Triangular Fuzzy Numbers. Math. Probl. Eng..

[79] Nădăban S., Dzitac S., Dzitac I. (2016). Fuzzy TOPSIS: A General View. Procedia Comput. Sci..

[80] Amer M., Daim T.U. (2011). Selection of renewable energy technologies for a developing county: A case of Pakistan. Energy Sustain. Dev..

[81] Lee H.C., Chang C.-T. (2018). Comparative analysis of MCDM methods for ranking renewable energy sources in Taiwan. Renew. Sustain. Energy Rev..

[82] Rahman S.M.S., Azeem A., Ahammed F. (2017). Selection of an appropriate waste-to-energy conversion technology for Dhaka City, Bangladesh. Int. J. Sustain. Eng..

[83] Sharholy M., Ahmad K., Mahmood G., Trivedi R. (2008). Municipal solid waste management in Indian cities—A review. Waste Manag..

[84] Özeler D., Yetiş Ü., Demirer G. (2006). Life cycle assesment of municipal solid waste management methods: Ankara case study. Environ. Int..

[85] Galgani P., van der Voet E., Korevaar G. (2014). Composting, anaerobic digestion and biochar production in Ghana. Environmental–economic assessment in the context of voluntary carbon markets. Waste Manag..

[86] Cudjoe D., Nketiah E., Obuobi B., Adu-Gyamfi G., Adjei M., Zhu B. (2021). Forecasting the potential and economic feasibility of power generation using biogas from food waste in Ghana: Evidence from Accra and Kumasi. Energy.

[87] Mohammed M., Egyir I., Donkor A., Amoah P., Nyarko S., Boateng K., Ziwu C. (2017). Feasibility study for biogas integration into waste treatment plants in Ghana. Egypt. J. Pet..

[88] Tyagi V.K., Lo S.-L. (2013). Sludge: A waste or renewable source for energy and resources recovery?. Renew. Sustain. Energy Rev..

[89] Munir M., Mardon I., Al-Zuhair S., Shawabkeh A., Saqib N. (2019). Plasma gasification of municipal solid waste for waste-to-value processing. Renew. Sustain. Energy Rev..

[90] Alao M.A., Popoola O.M., Ayodele T.R. (2021). Selection of waste-to-energy technology for distributed generation using IDOCRIW-Weighted TOPSIS method: A case study of the City of Johannesburg, South Africa. Renew. Energy.

[91] Mabalane P.N., Oboirien B.O., Sadiku E.R., Masukume M. (2021). A Techno-economic Analysis of Anaerobic Digestion and Gasification Hybrid System: Energy Recovery from Municipal Solid Waste in South Africa. Waste Biomass Valorization.

